# Pharmacological manipulation of TRPC5 by kaempferol attenuates metastasis of gastrointestinal cancer via inhibiting calcium involved in the formation of filopodia

**DOI:** 10.7150/ijbs.87829

**Published:** 2024-09-16

**Authors:** Suyun Yu, Rui Deng, Wei Wang, Defang Zou, Liang He, Zhonghong Wei, Yanhong Pan, Xiaoman Li, Yuanyuan Wu, Aiyun Wang, Wenxing Chen, Yang Zhao, Yin Lu

**Affiliations:** 1 Department of Biochemistry and Molecular Biology, School of Medicine & Holistic Integrative Medicine, Nanjing University of Chinese Medicine, Nanjing 210023, China.; 2 Jiangsu Collaborative Innovation Center of Traditional Chinese Medicine (TCM) Prevention and Treatment of Tumor, Nanjing University of Chinese Medicine, Nanjing 210023, China.; 3 Jiangsu Key Laboratory for Pharmacology and Safety Evaluation of Chinese Materia Medica, School of Pharmacy, Nanjing University of Chinese Medicine, Nanjing 210023, China.

**Keywords:** kaempferol, TRPC5, gastrointestinal cancer, calcium influx, filopodia

## Abstract

The thermo-sensory receptor, transient receptor potential channel 5 (TRPC5), a non-selective calcium ion (Ca^2+^)-permeable ion channel, has been implicated in cancer initiation and progression. However, its specific role in gastrointestinal cancer remains unclear. This study demonstrates that TRPC5 is significantly overexpressed in gastrointestinal tumors and is inversely associated with patient prognosis. TRPC5 overexpression triggers a substantial elevation in intracellular Ca^2+^ levels ([Ca^2+^]i), driving actin cytoskeleton reorganization and facilitating filopodia formation. Furthermore, kaempferol, a compound sourced from traditional Chinese medicine, is identified as a TRPC5 inhibitor that effectively suppresses its activity, thereby impeding gastrointestinal cancer metastasis. These findings underscore the potential of TRPC5 as a therapeutic target for metastasis inhibition, with kaempferol emerging as a promising natural inhibitor that could be optimized for clinical use in preventing cancer metastasis.

## Introduction

Gastrointestinal cancers, including gastric and colorectal cancers, rank among the most prevalent and deadly malignancies worldwide, as highlighted by the Global Cancer Statistics 2020 1. These cancers are characterized by rapid proliferation and aggressive invasion of nearby tissues and organs, with metastasis being the primary cause of mortality 2, 3. Despite advancements in understanding the mechanisms driving gastrointestinal cancer and the development of therapeutic and preventive measures, metastasis continues to pose a significant risk to patient survival. This underscores the urgent need to investigate critical molecular mechanisms and develop new intervention strategies targeting cancer metastasis.

Cancer cell motility is widely recognized as a fundamental process facilitating metastasis 4. Filopodia, actin-rich, finger-like protrusions extending from the plasma membrane, play a pivotal role in enhancing cell migration and invasion, processes strongly associated with cancer progression 5-7. Myosin-X (MYO10), a homodimeric molecular motor, is critical for promoting cancer cell invasion by inducing filopodia formation 8. Additionally, calcium channels have been implicated in the development and stabilization of filopodia, further promoting cancer metastasis 9, 10. Moreover, Ca^2+^ has been shown to influence the differentiation of various cell types, including those in the gastrointestinal tract 11. While these results suggest a link between Ca^2+^ and filopodia formation, the precise mechanisms by which Ca^2+^ influx drives gastrointestinal cancer metastasis remain elusive.

Thermo-sensory transient receptor potential channels (thermo-TRPs), a subset of non-selective Ca^2+^ channels, have been closely linked to cancer cell proliferation, migration, invasion, and angiogenesis, contributing to cancer progression and resistance to chemotherapy 12-15. These channels are activated within distinct temperature ranges, such as TRPV1 by temperatures above 43°C and TRPM8 by cold between 15°C and 28°C. TRPV1-4 and TRPM2-5 exhibit functional overlaps across a broad thermal spectrum from warm to hot, while TRPA1, TRPM8, and TRPC5 are sensitive to cooler temperatures 16-19. Among these cold-sensitive channels, TRPC5 has been particularly associated with the initiation and progression of various cancers. It facilitates the rapid influx of cations, primarily calcium ions, into the cytoplasm, maintaining osmotic pressure stability and mediating critical functions such as signal transduction 20-22. Although abnormal TRPC5 expression has been linked to cancer progression and chemoresistance, its role in regulating gastrointestinal cancer metastasis remains unexplored.

In addition to temperature activation, thermo-TRPs can be triggered by a wide array of small-molecule compounds. Many of these compounds, extracted from traditional Chinese medicine (TCM), such as capsaicin and menthol, possess "hot" or "cold" properties, inducing a warming or cooling sensation in the human body 23. Increasing evidence suggests that compounds with these thermal properties regulate thermogenesis through their interactions with TRPs. For instance, capsaicin and menthol, classic examples of "hot" and "cold" compounds, are natural agonists of TRPV1 and TRPM8, respectively 24. Other thermo-TRPs are similarly influenced by various compounds with temperature-associated properties. For example, TRPA1, TRPV3, and TRPV4 have been shown to be modulated by allicin, thymol, and cinnamaldehyde, respectively 25-27. Both literature 28 and our findings demonstrate that kaempferol, the primary component of *Rhizoma Kaempferiae*, a well-known TCM with warming properties, significantly inhibits TRPC5-mediated calcium influx and its downstream effects. Kaempferol has also been identified as a promising candidate for cancer prevention and treatment. Approximately 2.5% of ingested kaempferol is excreted in urine, while the majority is found in plasma and tissues at nanomolar concentrations 29, 30. Our research further indicates that kaempferol plays a critical role in inhibiting gastrointestinal cancer metastasis.

In this study, TRPC5 is identified as a key factor in counteracting the metastasis of gastrointestinal cancer, suggesting that targeted inhibition of TRPC5 in cancer cells may present a viable therapeutic strategy. Bioinformatics analyses, supported by extensive *in vitro* and *in vivo* experiments, reveal a negative correlation between TRPC5 expression and the progression of gastrointestinal cancer, positioning TRPC5 as a potential therapeutic target. Mechanistic investigations further demonstrate that TRPC5 promotes filopodia formation in gastrointestinal cancer cells *via* the ATP/p-MLC/p-cortactin signaling axis, unveiling a novel pathway driving cancer metastasis. The active ingredient kaempferol, derived from *Rhizoma Kaempferiae*, acts as a natural TRPC5 inhibitor and has shown efficacy in suppressing cancer metastasis, laying the groundwork for its potential development as an anti-metastasis agent for clinical application.

## Materials and methods

### Agents and Antibodies

Kaempferol (K107145) and GdCl_3_ (G119221) were sourced from Aladdin, while ML-204 was obtained from Med Chem Express (HY-12949). The antibodies used for Western blot, immunofluorescence, and immunohistochemistry included TRPC5 (Alomone, ACC-020), TRPC5 (Proteintech, 25890-1-AP), Myo-10 (Abcam, GR3199421-1), Akt (Cell Signaling Technology, 9272), p-Akt (Cell Signaling Technology, 4058), mTOR (SAB, 21214), PI3K (Proteintech, 21890-1-AP), CDC42 (Cell Signaling Technology, 2466), RhoA (Cell Signaling Technology, 2117), Rac1/2/3 (Cell Signaling Technology, 2465), p-myosin light chain (p-MLC, Ser19) (Cell Signaling Technology, 3671T), p-cortactin (Tyr421) (Affinity, AF3438), GAPDH (Bioworld), goat anti-rabbit IgG (H+L) HRP (Bioworld, AA86182), and goat anti-rabbit IgG H&L (FITC) (Abcam, Ab6717).

### Animals

BALB/c nude mice (6-8 weeks old, 18-22 g) were obtained from Charles River (Beijing, China) and housed under controlled conditions with a 12:12 light-dark cycle, a temperature of 21-23°C, humidity of 55%±10, and a standard diet.

### Mouse models of metastasis and therapeutic regimens

To create experimental metastasis models, MKN-45, DLD-1, red fluorescent protein (RFP)-tagged BGC-823 (BGC-823-RFP), TRPC5-silenced MKN-45 (MKN-45^TRPC5-/-^), and TRPC5-silenced DLD-1 (DLD-1^TRPC5-/-^) cells were injected into BALB/c nude mice. For the colorectal spleen injection liver metastasis model, the spleens were exposed for direct intrasplenic injection of DLD-1 cells suspended in 1 × PBS with 1mM EDTA. MKN-45 and BGC-823-RFP cells were administered intravenously. In the treatment protocols, ML-204 (10 mg/kg and 50 mg/kg), kaempferol (50 mg/kg and 100 mg/kg), or vehicle were intraperitoneally injected after tumor cell administration. Thirty days post-injection, the mice were euthanized, and metastatic nodules were counted using a stereoscopic microscope (Zeiss, Stemi 2000-C, Germany). Pulmonary metastasis following intraperitoneal injection of BGC-823-RFP cells was assessed through fluorescence intensity using the IVIS^TM^ Spectrum *in vivo* imaging system (PerkinElmer, IVIS Lumina III, USA).

For the orthotopic models of gastric and colorectal cancers, BALB/c nude mice were anesthetized with isoflurane, and MKN-45 and DLD-1 cells were orthotopically injected using sterile surgical techniques. After surgery, wounds were sutured and disinfected, with penicillin (20,000 U) administered intramuscularly to prevent infection. Thirty days post-injection, mice were euthanized and tissues were collected for further analysis.

### Histological assessment and Immunohistochemical (IHC) staining

For histological evaluation, tissue samples were fixed in 4% paraformaldehyde (PFA) overnight. The paraffin-embedded tissues were sectioned at a thickness of 4 µm using a microtome. Afterward, the sections were mounted onto slides, deparaffinized, and rehydrated. Hematoxylin and eosin (H&E) staining was performed as previously described 8.

For IHC analysis, tissue preparation followed the same process as histological assessment. This was followed by tissue blocking (Pierce, Rockford, IL, USA), primary antibody incubation overnight at 4°C, secondary antibody incubation, and Avidin-Biotin Complex incubation (Solarbio, Beijing, China). Images were captured using a microscope (PerkinElmer, Mantra^TM^, USA) and analyzed with ImageJ software (National Institutes of Health, USA).

### Quantitative real-time PCR

RNA extraction from the cells was carried out using TRIzol (Thermo Fisher Scientific, USA). Complementary DNA (cDNA) was synthesized from 500 ng of total RNA using Hiscript® II QRTSuperMix (Vazyme, Nanjing, China). Quantitative real-time PCR was performed using an SYBR Green Master kit (Vazyme, Nanjing, China), and the relative mRNA expression was quantified using the 2^(-ΔΔCt)^ method, normalized to GAPDH. The primers used for PCR are listed in Supplementary Table S1.

### Western blot analysis

For protein extraction, cells were lysed in ice-cold radioimmunoprecipitation assay (RIPA) buffer, and total protein extracts were separated *via* SDS-PAGE and then transferred onto polyvinylidene difluoride (PVDF) membranes. After blocking with bovine serum albumin (BSA) for 1 hour at room temperature, the membranes were incubated overnight at 4°C with the specified primary antibodies (diluted 1:500 - 1:2000). Following three PBS washes, the membranes were incubated with fluorophore-conjugated secondary antibodies (1:10000 dilution) for 2 hours at room temperature. The membranes were then washed three times with PBS and scanned using a Bio-Rad ChemiDoc XRS+ Imaging System (Hercules, China).

### Immunofluorescence (IF)

For immunofluorescence analysis, cells were seeded onto coverslips in 6-well plates and cultured in a medium containing 10% fetal bovine serum (FBS). Cancer cells were fixed with 4% PFA for 10 minutes, then blocked with 5% BSA for 30 minutes at room temperature. Cells were incubated with primary antibodies (1:50 - 1:200) overnight at 4°C, followed by incubation with fluorescent secondary antibodies for 2 hours at room temperature. Microfilaments were stained with Actin-tracker Green (C1033, C2205S, Beyotime, China), and nuclei were counterstained with DAPI (1 µg/mL) (C1006, Beyotime, China). Images were captured using a fluorescence microscope equipped with image processing and co-localization analysis software (Zeiss, Axio Vert.A1).

### Ca^2+^ influx measurement

Cells were seeded onto coverslips in 2 cm × 2 cm cell culture dishes and stained with Fura-2-acetoxymethyl ester (Fura-2-AM) in the presence of pluronic F-127 for 30 minutes at 37°C. After staining, the cells were washed three times with Hank's balanced salt solution (HBSS) containing 137 mM NaCl, 5.4 mM KCl, 1.3 mM CaCl_2_, 10 mM glucose, 20 mM HEPES, and 1 mM MgCl_2_ (adjusted to pH 7.4 with NaOH). The coverslips with cells were then placed in the perfusion chamber of a calcium imaging system, protected from light. Fluorescence images were captured using an Olympus fluorescence imaging system with dual excitation wavelengths of 340 and 380 nm. The ratio of emission signals at 340 nm and 380 nm excitation was used to calibrate and quantify the cytosolic Ca^2+^ concentration based on known free Ca^2+^ levels. Changes in the Fura-2 fluorescence ratios were calculated as alterations in intracellular [Ca^2+^]i.

### Transwell and wound healing migration assays

For the transwell migration assay, 1×10^5^ cancer cells, treated with dimethyl sulfoxide (DMSO) or varying concentrations of ML-204 (2, 4, 8 µM) or kaempferol (1, 2, 4, 8, 16, 32 µM), were seeded into the upper chamber of transwell inserts. Medium supplemented with 20%-30% FBS was added to the lower chamber, and the cells were incubated for 24 hours. After incubation, migrated cells on the lower surface of the upper chamber were stained with 0.1% crystal violet and visualized using an inverted bright-field microscope (Zeiss, AX10). Random fields were selected for each sample, and the number of migrated cells was counted.

In the wound healing assay, 5 × 10^5^ cells were seeded in 6-well plates and incubated at 37°C until confluent. Cells were then starved overnight in a serum-free medium, and a wound was created using a sterile 200 µL pipette tip. Floating cells were removed by rinsing with PBS, and a fresh medium containing DMSO, ML-204, or kaempferol at varying concentrations was added for 24 hours. The wound healing process was monitored and imaged at 0 and 24 hours using an inverted microscope (Zeiss, AX10). Cell migration was quantified as the percentage of wound closure.

### Three-dimensional (3D) invasion assay

For the 3D invasion assay, which was conducted as previously described 9, cancer cells stained with green fluorescent dye were seeded into Laser Confocal Petri Dishes (35 mm) and incubated at 37°C for 2-4 hours. Once the cells adhered, the supernatant was aspirated, and 300 µL of matrigel mix (basal medium: serum: matrix glue = 2:2:1), along with various concentrations of ML-204, kaempferol, or DMSO, was added. After 48 hours of incubation, images were captured using a Leica TCS SP5 Confocal microscope and analyzed with ImageJ software (National Institutes of Health, USA).

### Cell tracking

Tancer cell movement was monitored and captured using the Cell Observer Imaging System (Zeiss, Axio Vert.A1) over a 24-hour period with images taken at 20-minute intervals to track cell trafficking. After imaging, the images were converted into TIFF format and processed in grayscale mode. Individual cells were identified using Cell Tracker software, with "semi-automatic tracking" employed to specify the first and last images for analysis.

### Cellular thermal shift assay (CETSA)

Cells were washed with cold PBS and subjected to three freeze-thaw cycles using liquid nitrogen. The resulting lysates were aliquoted and incubated with DMSO or kaempferol for 30 minutes at room temperature. Each sample group was divided into twelve aliquots and heated to different temperatures (42°C, 44°C, 46°C, 48°C, 50°C, 52°C, 54°C, 56°C, 58°C, 60°C, 62°C, and 64°C) for 3 minutes, followed by cooling at room temperature for 3 minutes. The samples were then loaded onto SDS-PAGE gels and stained with Coomassie Brilliant Blue, followed by Western blot analysis as previously described.

### Drug affinity responsive target stability (DARTS) assay

Cells were washed with ice-cold PBS and lysed in a buffer containing protease and phosphatase inhibitors. After collection with a cell scraper, the cells were incubated on ice for 10 minutes. Following centrifugation at 18,000 × g for 10 minutes at 4°C, TNC buffer (Tris, NaCl, CaCl_2_) was added to the supernatants, and protein concentration was determined using the BCA Protein Assay Kit. The protein samples were divided into two parts equally and incubated with DMSO or kaempferol for 30 minutes at room temperature, then subjected to proteolysis at room temperature for 30 minutes using varying concentrations of pronase (1:100, 1:300, 1:1000, 1:3000, and 1:10000) (Roche Diagnostics, Indianapolis, USA). Pronase, a mixture of serine and acid proteases, is widely used in DARTS (Drug Affinity Responsive Target Stability) assays. Proteolysis was halted by EDTA addition, followed by a 10-minute incubation on ice. All samples were loaded onto SDS-PAGE gels, stained with Coomassie Brilliant Blue, and then analyzed *via* Western blot as described.

### Database access and analysis

The Kaplan Meier plotter database (http://www.kmplot.com/analysis/) is an online resource designed to evaluate the impact of 54,000 genes on cancer survival. In this study, the Kaplan Meier plotter was utilized to assess the prognostic significance of TRPC5 in gastric and colorectal cancer patients. Hazard ratios (HRs) with 95% confidence intervals and log-rank p-values were calculated, with statistical significance set at p < 0.05.

The Linked Omics database (http://www.linkedomics.org/login.php) is a public platform that hosts multi-omics data from 32 TCGA cancer types and 10 Clinical Proteomics Tumor Analysis projects. Differentially expressed genes related to TRPC5 were identified from stomach adenocarcinoma (STAD) and colon adenocarcinoma (COAD) using the LinkFinder module, and results were validated through Pearson correlation coefficient analysis.

The TRansient receptor potential channel-Interacting Protein (TRIP) database (http://trpchannel.org) is a curated repository of protein-protein interactions specific to mammalian TRPs. This study extracted TRPC5 protein interaction data from the TRIP database, which was subsequently imported into Cytoscape software (http://www.cytoscape.org) for network visualization after downloading the data in MS Excel or sif formats.

### Ethics statement

All animal experiments were conducted in accordance with ethical guidelines and procedures approved by the ethics committee of Nanjing University of Chinese Medicine (Approval no. ACU170502). Informed consent was obtained from all human patients providing clinical samples.

### Statistical analysis

Data were presented as the mean ± SD from at least three independent experiments. Statistical analyses were carried out using GraphPad Prism version 7.0 (GraphPad Software, San Diego, USA). Group comparisons were made using one-way analysis of variance (ANOVA), with p < 0.05 considered statistically significant.

## Results

### TRPC5 is functionally overexpressed in gastrointestinal cancer and negatively correlated with patient prognosis

**Figure 1:**
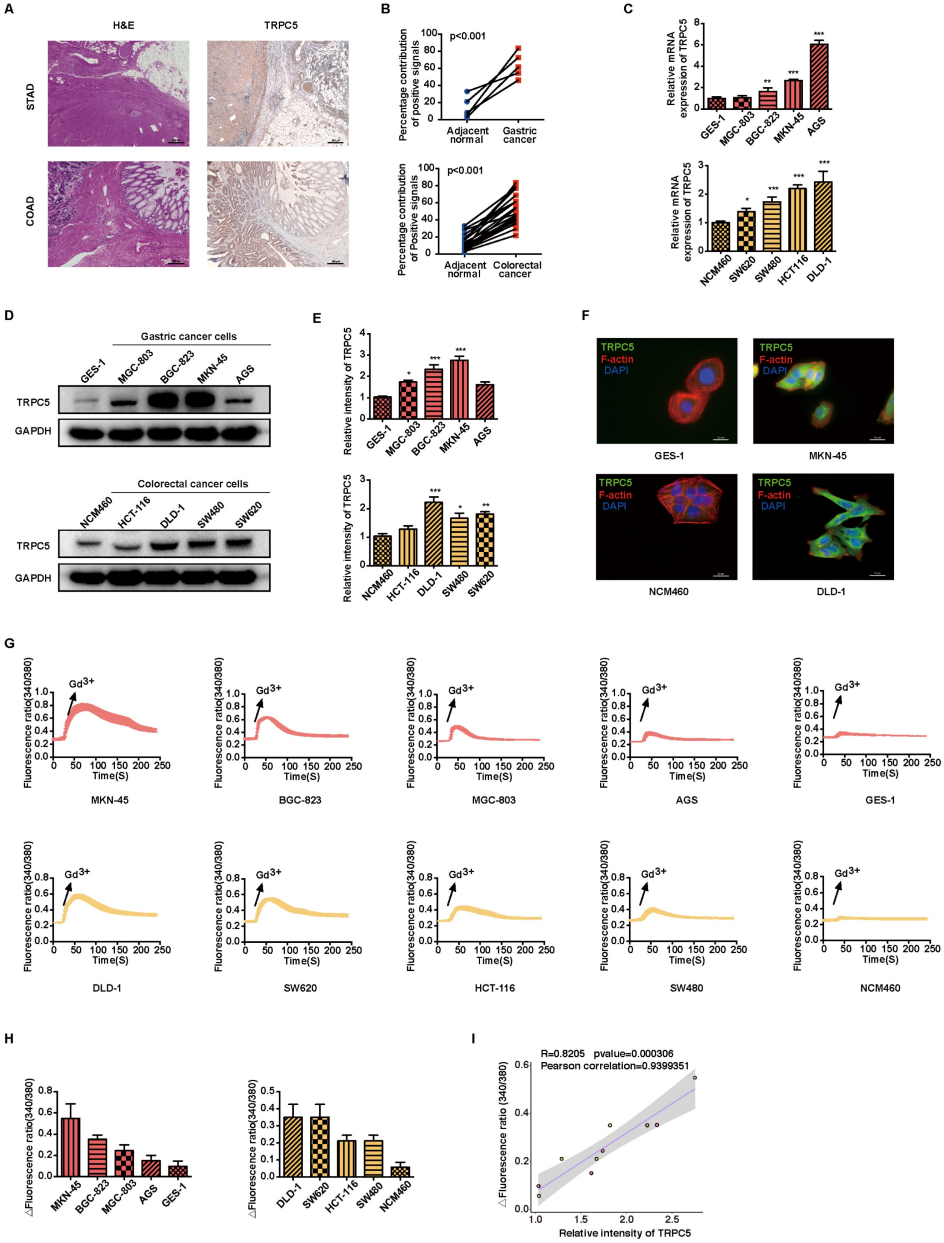
TRPC5 is functionally overexpressed in gastrointestinal cancer. (A) Representative HE and TRPC5 IHC staining images from clinical gastric and colorectal cancer samples (n = 5 for STAD, n = 13 for COAD). Scale bar: 200 μm. (B) Quantitative analysis of TRPC5 IHC staining levels between tumors and adjacent normal tissues (P < 0.001 vs. adjacent normal tissues). (C) Relative TRPC5 mRNA expression in indicated gastric and colorectal cancer cells. Data are expressed as means ± SD, *P < 0.05, **P < 0.01, ***P < 0.001 (vs. GES-1 or NCM460 cells). (D, E) Immunoblot analysis of TRPC5 protein expression in the indicated gastric and colorectal cancer cells. Data are expressed as means ± SD, *P < 0.05, **P < 0.01, ***P < 0.001 (vs. GES-1 or NCM460 cells). (F) Representative IF staining of TRPC5 in GES-1, MKN-45, NCM460, and DLD-1 cells (blue: nuclei, red: F-actin, green: TRPC5). Scale bar: 50 μm. (G) Averaged time courses of Ca^2+^ imaging (F340/380) and representative traces of calcium responses in gastric and colorectal cancer cells induced by 50 μM Gd^3+^. (H) Maximal [Ca^2+^]i increases (ΔF340/380) in gastric and colorectal cancer cells evoked by 50 μM Gd^3+^ (n = 10). Data are expressed as means ± SD. (I) The Pearson correlation analysis revealed a strong linear relationship between the expression of TRPC5 and the maximal rise in intracellular Ca2+ concentration (ΔF340/380), with a correlation coefficient of 0.9399351.

To investigate the significance of TRPC5 expression in gastrointestinal cancer, both pathological and TRPC5 immunohistochemical staining were conducted on clinical samples of STAD and COAD, along with matched adjacent normal tissues (Figure 1A). The analysis revealed a significant upregulation of TRPC5 expression in tumor tissues compared to adjacent normal tissues (Figure 1A-1B and Supplementary Table S2). Consistent with these findings, Kaplan-Meier survival curves indicated a negative correlation between TRPC5 expression and the prognosis of patients with gastric and colorectal cancers (Supplementary Figure 1A). Additionally, compared to normal gastric epithelial cells (GES-1) and colon epithelial cells (NCM460), TRPC5 mRNA and protein levels were markedly elevated in gastric cancer cell lines (MGC-803, BGC-823, MKN-45, AGS) and colorectal cancer cell lines (HCT-116, DLD-1, SW480, SW620) (Figure 1C-1E). Notably, MKN-45 and DLD-1 exhibited the highest TRPC5 protein expression among the respective gastric and colorectal cancer cell lines (Figure 1D-1E), leading to their selection for subsequent *in vitro* and *in vivo* studies. Supporting these observations, IF staining in cancer cells (Figure 1F) and IHC staining in primary tumors (Supplementary Figure 1B) confirmed significantly elevated TRPC5 protein levels in gastrointestinal cancer cells compared to normal cells. Given that TRPC5 functions as a non-selective Ca^2+^ channel, intracellular calcium mobilization serves as a critical indicator of TRPC5 activation, subsequently triggering a range of downstream physiological and pathological processes 31. To evaluate the correlation between TRPC5 activation and Ca^2+^ influx in gastrointestinal cancer cells, Ca^2+^ fluorescence imaging was performed. As demonstrated in Figure 1G, Fura-2-AM imaging revealed that TRPC5 activation induces a rapid and sustained intracellular Ca^2+^ influx in gastrointestinal cancer cells, with the extent of intracellular Ca^2+^ increase positively correlating with TRPC5 expression levels (Figure 1H-1I). Collectively, these results suggest that TRPC5 is functionally overexpressed in gastrointestinal cancer cells and likely contributes to cancer progression.

### TRPC5 overexpression exacerbates the metastasis of gastrointestinal cancer

**Figure 2:**
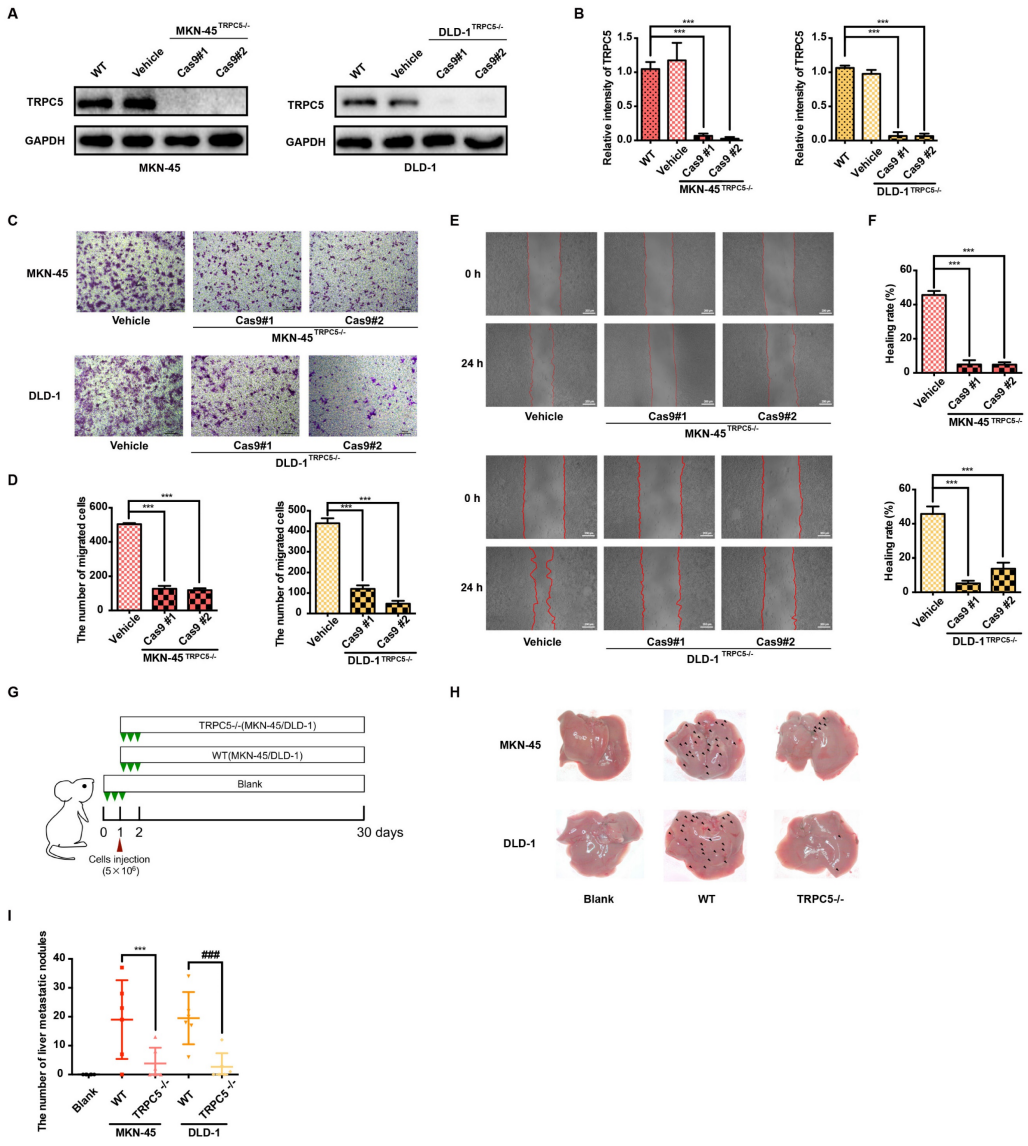
Deletion of TRPC5 attenuates gastrointestinal cancer metastasis. (A, B) Immunoblot analysis of TRPC5 protein expression in MKN-45 and DLD-1 cells modified by the CRISPR/Cas9 system. Data are expressed as means ± SD, ***P < 0.001 (vs. WT group). (C) Representative images from the transwell migration assay showing the migration of MKN-45 and DLD-1 cells treated with vehicle control or TRPC5 knockout (MKN-45^TRPC5-/-^ and DLD-1^TRPC5-/-^). Random visual fields were selected. Scale bar: 500 μm. (D) Bar graph of migrated MKN-45 and DLD-1 cells in vehicle control and TRPC5 knockout groups. Data are expressed as means ± SD, ***P < 0.001 (vs. vehicle group). (E) Representative images from the wound healing assay in vehicle control and TRPC5 knockout groups. (F) Healing rates of MKN-45 and DLD-1 cells in vehicle control and TRPC5 knockout groups. Data are expressed as means ± SD, ***P < 0.001 (vs. vehicle group). (G) Schematic of animal study: 5 × 10^6^ MKN-45 and DLD-1 (WT or TRPC5 knockout) cells were injected into BALB/c nude mice, and metastasis was assessed 30 days post-injection (n = 6 per group). (H, I) Analysis of liver metastasis in mice engrafted with MKN-45 and DLD-1 cells (WT or TRPC5 knockout) at day 30. Data are expressed as means ± SD, ***P < 0.001 (vs. MKN-45^WT^ group), ^###^P < 0.001 (vs. DLD-1^WT^ group).

Metastasis remains the leading cause of mortality in cancer patients, with approximately 80% of those with malignant tumors dying as a result of metastasis 32. Gastrointestinal cancer, characterized by poor prognosis and high mortality, sees metastasis as a primary contributor to patient deaths 33. Thus, an in-depth investigation into gastrointestinal cancer metastasis is essential for advancing treatment strategies. To explore the role of TRPC5 in modulating metastasis, TRPC5-specific knockout (TRPC5^-/-^) MKN-45 and DLD-1 cell lines were successfully generated using CRISPR-Cas9 technology (Figure 2A-2B, Supplementary Figure 2A). To assess whether TRPC5 expression impacts cancer cell migration, transwell and wound healing assays were conducted *in vitro*. The findings revealed a significant reduction in the migratory and invasive capabilities of TRPC5^-/-^ cells compared to control cells (Figure 2C-2F). Additionally, wild-type (WT) and TRPC5-silenced MKN-45 and DLD-1 cells were injected into BALB/c nude mice, and metastatic nodules on liver surfaces were analyzed 30 days post-injection (Figure 2G). Notably, mice injected with WT cells exhibited a higher number of metastatic nodules on liver surfaces compared to those injected with TRPC5^-/-^ cells (Figure 2H-2I), with no significant difference between the empty vector and WT groups (Supplementary Figure 2B-2C). These results suggest that TRPC5 plays a critical role in promoting gastrointestinal cancer metastasis.

### Pharmacological inhibition of TRPC5 attenuates the metastasis of gastrointestinal cancer

**Figure 3:**
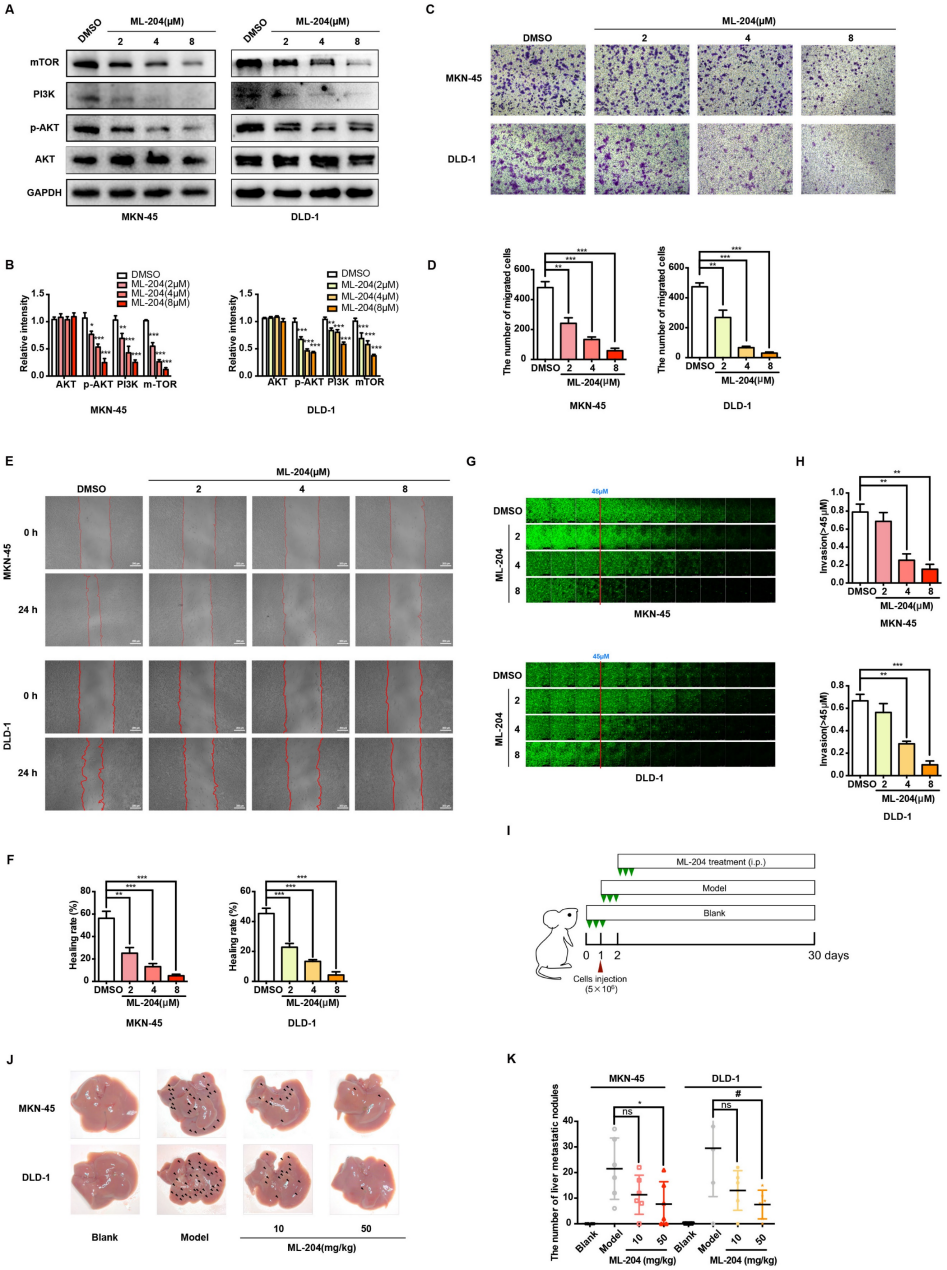
Functional TRPC5 is critical for gastrointestinal cancer metastasis. (A, B) Immunoblot analysis of AKT, p-AKT, PI3K, and mTOR expression in MKN-45 and DLD-1 cells treated with ML-204 (2, 4, and 8 μM). Data are expressed as means ± SD, *P < 0.05, **P < 0.01, ***P < 0.001 (vs. DMSO group). (C) Representative images of MKN-45 and DLD-1 cell migration treated with ML-204 (2, 4, and 8 μM) in the transwell migration assay. Random visual fields were selected. Scale bar: 500 μm. (D) Bar graph showing the number of migrated MKN-45 and DLD-1 cells after ML-204 treatment (2, 4, and 8 μM). Data are expressed as means ± SD, **P < 0.01, ***P < 0.001 (vs. DMSO group). (E) Representative wound healing assay images of MKN-45 and DLD-1 cells treated with ML-204 (2, 4, and 8 μM). (F) Healing rates of MKN-45 and DLD-1 cells after ML-204 treatment. **P < 0.01, ***P < 0.001 (vs. DMSO group). (G) Inverted invasion assay evaluating MKN-45 and DLD-1 cell invasion in the presence of ML-204 (2, 4, and 8 μM). Scale bar: 250 μm. (H) Quantification of relative invasion abilities of MKN-45 and DLD-1 cells over 45 μm (n = 3). Data are expressed as means ± SD, **P < 0.01, ***P < 0.001 (vs. DMSO group). (I) Schematic of animal study: 5 × 10^6^ MKN-45 or DLD-1 cells were injected into BALB/c nude mice, followed by intraperitoneal injection of ML-204 (10 mg/kg and 50 mg/kg) starting on day 2 post-injection (n = 8 per group). (J, K) Liver metastasis analysis after engraftment of MKN-45 or DLD-1 cells. Data are expressed as means ± SD, ^*^P < 0.05 (vs. MKN-45 model group), ^#^P < 0.05 (vs. DLD-1 model group).

To further elucidate the functional role of TRPC5, the selective TRPC5 channel inhibitor ML-204 34 was utilized to assess its effects on gastrointestinal cancer metastasis. Initially, the impact of ML-204 on cancer cell proliferation was evaluated, and concentrations that did not affect cell proliferation were selected for further investigation (data not shown). ML-204 was shown to significantly reduce intracellular Ca^2+^ influx, confirming its inhibitory effect on TRPC5 function (Supplementary Figure 3A-3B). Correspondingly, the expression of mTOR, PI3K, and p-AKT, key downstream targets of Ca^2+^ influx, was markedly suppressed following ML-204 treatment (Figure 3A-B). Consistent with observations in TRPC5-silenced cancer cells, ML-204 significantly inhibited the migration and invasion of MKN-45 and DLD-1 cells, as demonstrated by transwell, wound healing, and 3D invasion assays (Figure 3C-3H). Notably, similar outcomes were observed in the xenograft model, where mice in the model group exhibited pronounced liver metastasis 30 days post-injection of cancer cells, while those treated with ML-204 showed a marked reduction in liver metastatic nodules (Figure 3I-3J). Statistical analysis revealed no significant difference in metastatic nodules between the vehicle-treated group and the 10 mg/kg ML-204 group, while 50 mg/kg ML-204 led to a significant reduction in metastatic nodules compared to vehicle control (Figure 3K). In summary, these results confirm that functional inhibition of TRPC5 by ML-204 effectively attenuates gastrointestinal cancer metastasis.

### TRPC5 affects metastasis of gastrointestinal cancer by regulating filopodia formation

**Figure 4:**
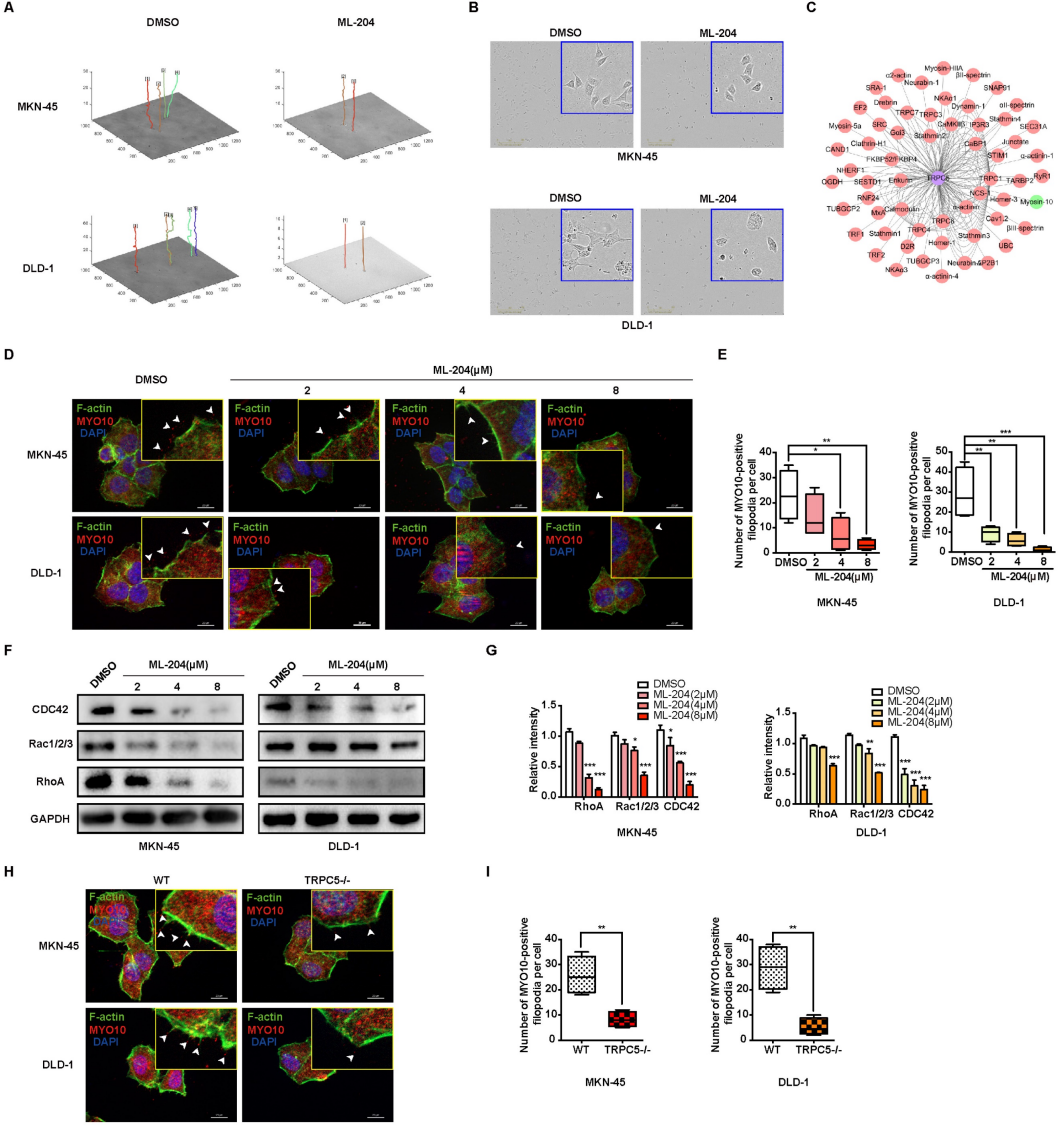
TRPC5 promotes the motility and filopodia formation in gastrointestinal cancer cells. (A) Schematic representation of the movement trajectory in MKN-45 and DLD-1 cells treated with ML-204 (8 μM). (B) Representative bright-field images of MKN-45 and DLD-1 cells after treatment with ML-204 (8 μM). Scale bar: 300 μm. (C) TRPC5 protein-protein interaction network based on the TRIP database. (D) Representative MYO10 immunofluorescence (IF) staining in MKN-45 and DLD-1 cells following treatment with ML-204 (2, 4, and 8 μM) (blue: nuclei, red: MYO10, green: F-actin). Scale bar: 20 μm. (E) Quantification of MYO10-positive filopodia in MKN-45 and DLD-1 cells treated with ML-204 (2, 4, and 8 μM). Data are expressed as means ± SD, *P < 0.05, **P < 0.01, ***P < 0.001 (vs. DMSO group). (F, G) Immunoblot analysis of RhoA, Rac1/2/3, and CDC42 expression in MKN-45 and DLD-1 cells after ML-204 treatment (2, 4, and 8 μM). Data are expressed as means ± SD, *P < 0.05, **P < 0.01, ***P < 0.001 (vs. DMSO group). (H) Representative MYO10 IF staining in MKN-45 and DLD-1 cells in WT and TRPC5 knockout groups (blue: nuclei, red: MYO10, green: F-actin). Scale bar: 20 μm. (I) Quantification of MYO10-positive filopodia in WT and TRPC5 knockout MKN-45 and DLD-1 cells. Data are expressed as means ± SD, **P < 0.01 (vs. WT group).

The mechanisms by which TRPC5 influences gastrointestinal cancer cell metastasis were further explored. Increasing evidence suggests that intracellular calcium ion fluctuations play a pivotal role in cell apoptosis 35. Flow cytometry analysis, however, reveals that TRPC5 inhibition exerts no significant effect on cancer cell apoptosis (Supplementary Figure 4A-4B). To investigate the underlying mechanisms, the LinkedOmics database (http://www.linkedomics.org/login.php) was utilized to identify genes potentially associated with TRPC5 in STAD and COAD 36. Gene Ontology analysis further shows that TRPC5-related genes are positively linked to motile cilium, actinin binding, cytoskeletal organization, and other motility-related functions (Supplementary Figure 4C-4D), suggesting a role for TRPC5 in regulating cell movement. Live-cell imaging combined with Cell Tracker software demonstrated that TRPC5 inhibition by ML-204 significantly restricts the motility of MKN-45 and DLD-1 cells, with the movement trajectories in the ML-204-treated group appearing notably flatter compared to those in the DMSO-treated group (Figure 4A). It is well-known that the formation of F-actin-rich protrusions is a hallmark of cell motility, facilitating cellular extension, adhesion, and propulsion, with invasive protrusions aiding matrix penetration 37, 38. Enlarged images of cell morphology confirm that the pseudopod-like structures in MKN-45 and DLD-1 cells are substantially disrupted following ML-204 treatment (Figure 4B). Using the Turning Research into Practice (TRIP) database and Cytoscape software for network visualization, TRPC5 was found to be preferentially associated with MYO10, a key marker of filopodia that interacts with multiple cytoskeletal proteins (Figure 4C). To determine whether TRPC5 modulates cancer cell motility *via* filopodia formation, we observed that ML-204 markedly suppresses MYO10-induced filopodia in MKN-45 and DLD-1 cells in a concentration-dependent manner (Figure 4D-4E). Small GTPases of the Rho family, including Cdc42, Rac, and Rho, are critical regulators of cell migration and filopodia formation 39. Interestingly, ML-204 also reduces the expression of CDC42, Rac1/2/3, and RhoA in a dose-dependent manner in MKN-45 and DLD-1 cells (Figure 4F-4G), consistent with the suppression of MYO10-induced filopodia in TRPC5^-/-^ cell lines (Figure 4H-4I). Collectively, these results suggest that TRPC5 channels promote filopodia formation in gastrointestinal cancer cells, thereby enhancing motility.

### TRPC5 boosts the phosphorylation of MLC to promote the formation of filopodia

**Figure 5:**
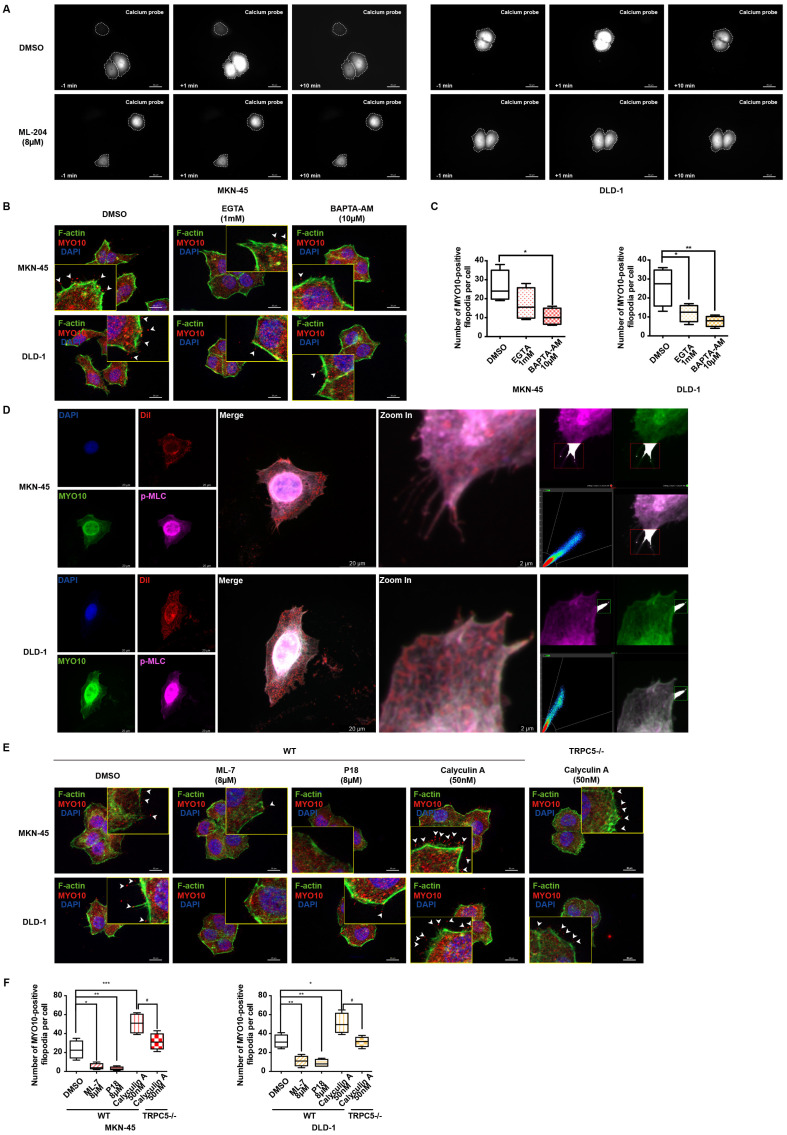
TRPC5 promotes filopodia formation *via* Ca^2+^-dependent MLC activation. (A) MKN-45 and DLD-1 cells expressing the calcium probe (GCaMP6s) were pretreated with DMSO or ML-204 (8 μM) before stimulation with Gd^3+^. The relative increase in intracellular calcium probe intensity was measured at 1 and 10 minutes post-stimulation, with cell boundaries indicated by dotted lines. Scale bar: 50 μm. (B) Representative MYO10 immunofluorescence (IF) staining in MKN-45 and DLD-1 cells treated with EGTA (1 mM) or BAPTA-AM (10 μM) (blue: nuclei, red: MYO10, green: F-actin). Scale bar: 20 μm. (C) Quantification of MYO10-positive filopodia in MKN-45 and DLD-1 cells treated with EGTA (1 mM) or BAPTA-AM (10 μM). Data are expressed as means ± SD, *P < 0.05, **P < 0.01 (vs. DMSO group). (D) Representative IF staining of MKN-45 and DLD-1 cells (blue: nuclei, red: dil, green: MYO10, violet: p-MLC). Scale bar: 20 μm (left). Zoomed-in images and fluorescence co-localization of p-MLC and MYO10 at filopodia tips in MKN-45 and DLD-1 cells (MKN-45: Pearson > 0.9; DLD-1: Pearson > 0.9). Scale bar: 2 μm (right). (E) Representative MYO10 IF staining of MKN-45 and DLD-1 cells treated with Calyculin A (50 nM), ML-7 (8 μM), or P18 (8 μM) (blue: nuclei, red: MYO10, green: F-actin). Scale bar: 20 μm. (F) Quantification of MYO10-positive filopodia in MKN-45 and DLD-1 cells treated with Calyculin A (50 nM), ML-7 (8 μM), or P18 (8 μM). Data are expressed as means ± SD, *P < 0.05, **P < 0.01, ***P < 0.001 (vs. DMSO group).

Extensive research has demonstrated that intracellular calcium ions bind to calmodulin (CaM), forming a Ca^2+^-CaM complex, which subsequently activates numerous calcium-dependent proteins 40, 41. As a genetically encoded fluorescent Ca^2+^ indicator, the GCaMP6s probe is utilized to monitor intracellular Ca^2+^-CaM complex dynamics. Live cell fluorescence imaging reveals a rapid escalation of the Ca^2+^-CaM complex following TRPC5 activation, an effect significantly diminished when cells are pre-incubated with ML-204, indicating that ML-204 impedes the TRPC5-induced formation of the Ca^2+^-CaM complex (Figure 5A). Additionally, both the extracellular calcium chelator EGTA (ethylene glycol tetraacetic acid) and the intracellular chelator BAPTA-AM (1,2-Bis(2-aminophenoxy)ethane-N,N,N,N'-tetraacetic acid tetrakis (acetoxymethyl ester)) effectively suppress MYO10-induced filopodia formation in MKN-45 and DLD-1 cells. Notably, BAPTA-AM exhibits a more pronounced inhibitory effect than EGTA, underscoring the critical role of intracellular calcium in enhancing filopodia production (Figure 5B-5C).

Previous studies have highlighted that pp60 c-src phosphorylates MLC kinase (MLCK), which, in turn, increases MLC phosphorylation following the formation of the Ca^2+^-CaM complex 42. It has been established that TRPC5 activation enhances MLC phosphorylation, an effect reversed when TRPC5 is inhibited by ML-204 (Supplementary Figure 5A). Furthermore, MLCK inhibition *via* ML-7 confirms that TRPC5-mediated MLC phosphorylation occurs through MLCK (Supplementary Figure 5A). To further examine the role of p-MLC in filopodia formation, colocalization analysis with MYO10 was conducted, revealing a significant colocalization of p-MLC with MYO10 at the tips of filopodia in both MKN-45 and DLD-1 cells (Figure 5D). Consistent with these findings, calyculin A, an activator of MLC phosphorylation, significantly promotes MYO10-induced filopodia formation, whereas ML-7 and P18, two classic MLCK inhibitors, substantially impede this process (Figure 5E-5F). Even in TRPC5^-/-^ cell lines, calyculin A continues to increase MYO10-induced filopodia formation to some extent, although this effect is markedly reduced compared to wild-type cells (Figure 5E-5F). Collectively, these results suggest that TRPC5 likely facilitates filopodia formation through Ca^2+^-dependent MLC activation.

### TRPC5 promotes the formation of filopodia through the ATP/p-MLC/p-cortactin signaling axis

**Figure 6:**
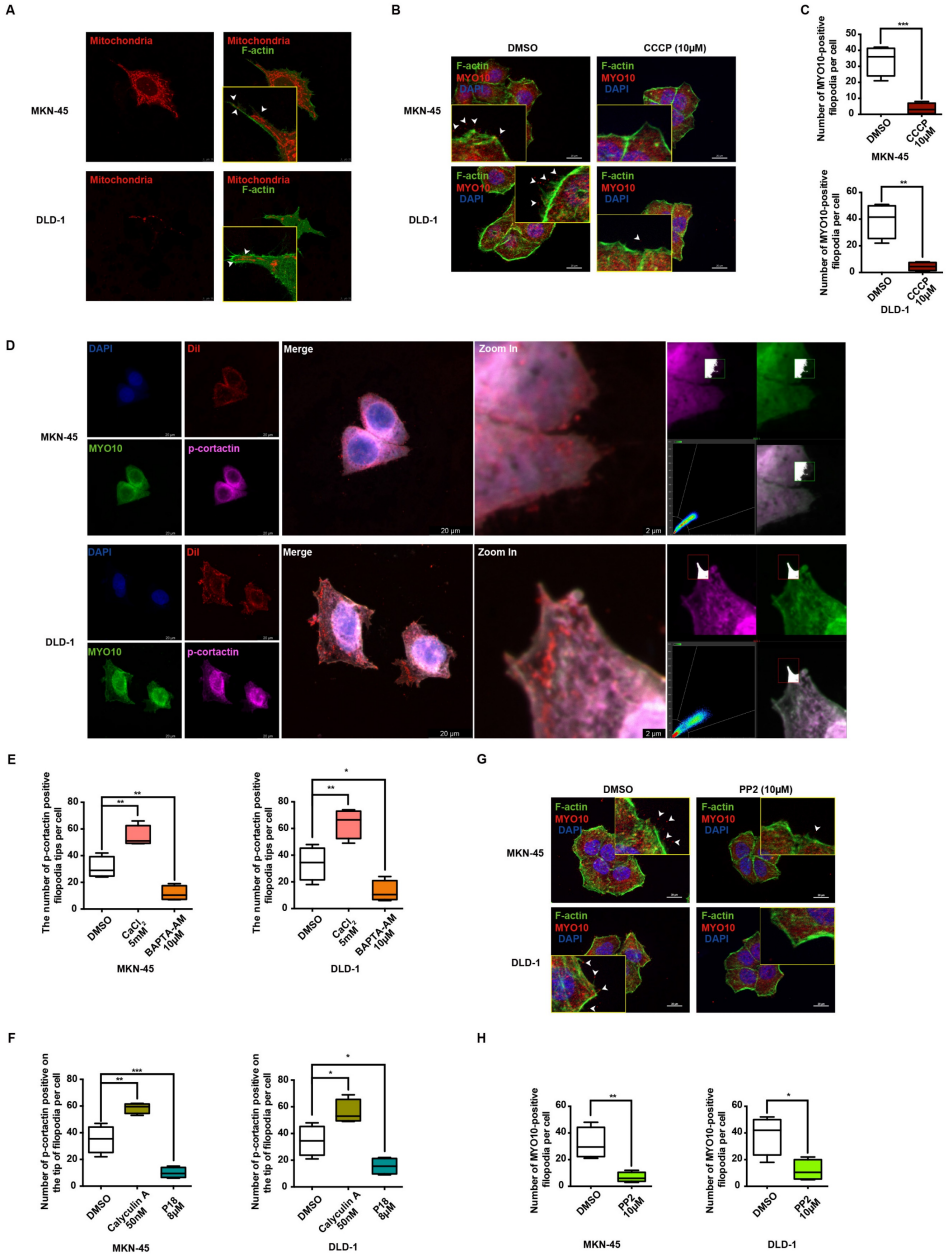
Ca^2+^-dependent MLC activation enhances filopodia formation *via* p-cortactin rearrangement. (A) Representative immunofluorescence (IF) images of MKN-45 and DLD-1 cells stained for mitochondria and F-actin (red: mitochondria, green: F-actin). Scale bar: 10 μm. (B) Representative MYO10 IF staining in MKN-45 and DLD-1 cells treated with CCCP (10 μM) (blue: nuclei, red: MYO10, green: F-actin). Scale bar: 20 μm. (C) Quantification of MYO10-positive filopodia in MKN-45 and DLD-1 cells treated with CCCP (10 μM). Data are expressed as means ± SD, **P < 0.01, ***P < 0.001 (vs. DMSO group). (D) Representative IF staining of MKN-45 and DLD-1 cells (blue: nuclei, red: dil, green: MYO10, violet: p-cortactin). Scale bar: 20 μm (left). Zoomed-in images and fluorescence co-localization of p-cortactin and MYO10 at filopodia tips in MKN-45 and DLD-1 cells (MKN-45: Pearson > 0.9; DLD-1: Pearson > 0.9). Scale bar: 2 μm (right). (E) Quantification of p-cortactin-positive filopodia tips per cell in MKN-45 and DLD-1 cells treated with CaCl_2_ (5 mM) or BAPTA-AM (10 μM). Data are expressed as means ± SD, *P < 0.05, **P < 0.01 (vs. DMSO group). (F) Quantification of p-cortactin-positive filopodia tips per cell in MKN-45 and DLD-1 cells treated with calyculin A (50 nM) or P18 (8 μM). Data are expressed as means ± SD, *P < 0.05, **P < 0.01, ***P < 0.001 (vs. DMSO group). (G) Representative MYO10 IF staining in MKN-45 and DLD-1 cells treated with PP2 (10 μM) (blue: nuclei, red: MYO10, green: F-actin). Scale bar: 20 μm. (H) Quantification of MYO10-positive filopodia in MKN-45 and DLD-1 cells treated with PP2 (10 μM). Data are expressed as means ± SD, *P < 0.05, **P < 0.01 (vs. DMSO group).

Numerous studies have increasingly indicated that MLC phosphorylation is dependent on ATP supply, which plays a pivotal role in regulating cellular dynamics 43. IF data reveal a marked increase in MLC phosphorylation levels in MKN-45 and DLD-1 cells upon stimulation with 10μM ATP (Supplementary Figure 5B). Mitochondria, as the cell's powerhouse, generate ATP by converting ADP, thereby providing energy for various physiological activities 44. Mito-Tracker Red CMXRos specifically labels bioactive mitochondria, and fluorescent co-staining shows mitochondrial localization predominantly at the filopodia axes and tips (Figure 6A). Additionally, carbonylcyanide3-chlorophenylhydrazone (CCCP), a potent oxidative phosphorylation uncoupler, significantly suppresses filopodia formation in gastrointestinal cancer cells (Figure 6B-6C), suggesting a strong association between ATP function and filopodia formation.

Cancer cell migration and invasion are known to depend on pseudopodium formation, which includes various subtypes based on morphology, with filopodia and lamellipodia being the primary ones 39. MYO10 and cortactin (CTTN) are key markers for filopodia and lamellipodia, respectively. Phosphorylation of cortactin facilitates its rapid membrane localization, enhancing pseudopodia invasion, and is previously reported to be activated by MLC phosphorylation 45. To further investigate the relationship between p-cortactin and filopodia, IF co-localization experiments were performed. Strikingly, the results reveal a clear co-localization of p-cortactin and MYO10 at filopodia tips in both MKN-45 and DLD-1 cells (Figure 6D). The regulation of cortactin phosphorylation by calcium ions and p-MLC was also explored. As shown in Figure 6E and Supplementary Figure 5C, the number of p-cortactin positive signals at filopodia tips per cell significantly increases upon exogenous CaCl_2_ addition, whereas the calcium chelator BAPTA-AM diminishes the p-cortactin signal. Correlation analysis in STAD and COAD tumors underscores a significant positive correlation between MYO10 and CTTN (Supplementary Figure 5D). Notably, the activation and expression of p-cortactin at filopodia tips appear to be regulated by p-MLC. Phosphorylation of MLC by calyculin A dramatically enhances p-cortactin signals at filopodia tips, while P18 reduces p-cortactin formation by inhibiting MLC phosphorylation (Figure 6F and Supplementary Figure 5E). PP2, a reversible ATP-competitive Src family inhibitor, effectively inhibits cortactin phosphorylation 46. Our findings indicate that PP2 significantly diminishes filopodia formation mediated by cortactin phosphorylation (Figure 6G-6H). Collectively, these data suggest that TRPC5 may promote filopodia formation by activating the ATP/p-MLC/p-Cortactin signaling axis.

### Kaempferol derived from TCM serves as a natural small molecule inhibitor of TRPC5

**Figure 7:**
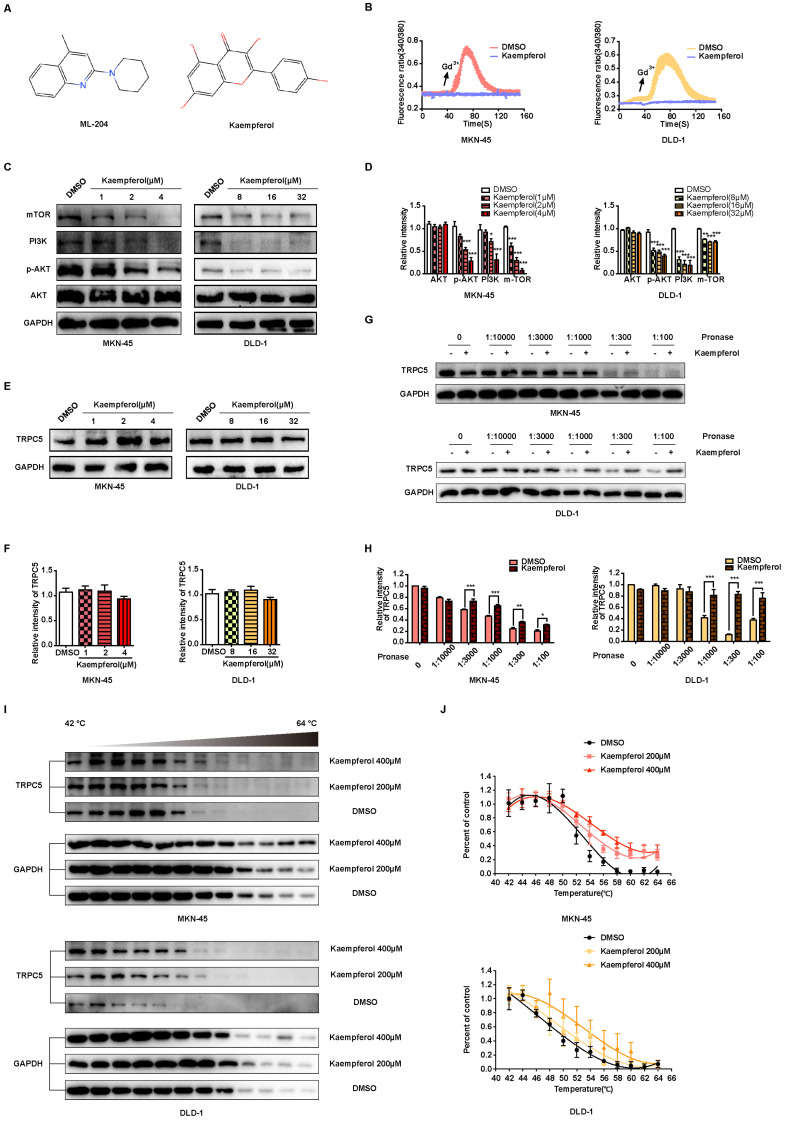
Kaempferol functions as a potent natural TRPC5 inhibitor. (A) Chemical structures of ML-204 and kaempferol. (B) Averaged Ca^2+^ imaging time courses (F340/380) with representative calcium response traces in MKN-45 and DLD-1 cells pretreated with or without kaempferol, following stimulation with 50 μM Gd^3+^. (C, D) Immunoblot analysis of AKT, p-AKT, PI3K, and mTOR expression in MKN-45 and DLD-1 cells treated with varying concentrations of kaempferol. Data are expressed as means ± SD, *P < 0.05, **P < 0.01, ***P < 0.001 (vs. DMSO group). (E, F) Immunoblot analysis of TRPC5 protein levels in MKN-45 and DLD-1 cells treated with different concentrations of kaempferol. Data are expressed as means ± SD. (G, H) DARTS detection *via* immunoblot in MKN-45 and DLD-1 cells. Jurkat cell lysates were incubated with kaempferol (200 μM) or DMSO, followed by digestion with Pronase at protein ratios of 1:10000, 1:3000, 1:1000, 1:300, and 1:100. Data are expressed as means ± SD, *P < 0.05, **P < 0.01, ***P < 0.001 (vs. DMSO group). (I, J) CETSA detection *via* immunoblot in MKN-45 and DLD-1 cells. Jurkat cell lysates were incubated with kaempferol (200 μM and 400 μM) or DMSO, followed by a 3-minute incubation at a temperature gradient of 42-64°C.

TCM is recognized for producing unique natural compounds in response to environmental stress, following the principles of species evolution. These compounds, which interact with biological targets, are noted for their structural diversity, low toxicity, and minimal side effects 47. According to Johnson and Maggiora's principle of molecular similarity, which states "similar compounds exhibit similar properties," DISCOVERY STUDIO 4.0 and relevant literature were employed to identify small molecule inhibitors of TRPC5 from TCM for potential anti-cancer therapy. Notably, kaempferol, derived from *Rhizoma Kaempferiae*, exhibits significant structural similarity to ML-204 (Figure 7A) and has been identified as a potential antagonist of TRPC5 function 28. In line with ML-204, non-proliferative concentrations of kaempferol for MKN-45 and DLD-1 cells were selected for further investigation (Data not shown). Notably, the Ca^2+^ influx triggered by Gd^3+^ was completely inhibited by kaempferol treatment (Figure 7B). Additionally, kaempferol substantially reduced the expression levels of mTOR, PI3K, and p-AKT, downstream effectors of Ca^2+^ influx (Figures 7C-7D). Moreover, thermo-TRPs, which can be activated by small molecules, are often responsive to compounds with “cold” or “hot” properties in TCM. Interestingly, *Rhizoma Kaempferiae* is classified as having a "hot" property, and its active component, kaempferol, might serve as a functional TRPC5 inhibitor.

Further investigation revealed that kaempferol did not alter TRPC5 protein levels in cancer cells (Figures 7E-7F). Given that TRPC5 is a selective Ca^2+^ channel, the next step was to assess whether kaempferol's effects were tied to its ability to bind TRPC5 and block Ca^2+^ channel activation. CETSA and DARTS, two label-free biophysical assays, were employed to evaluate the interaction between kaempferol and TRPC5 48-50. The results indicate that kaempferol enhances the proteolytic resistance of TRPC5 compared to the control (Figures 7G-7H). Additionally, kaempferol improved the thermal stability of TRPC5 (Figures 7I-7J). Collectively, these results demonstrate that kaempferol directly binds to TRPC5, thereby inhibiting its activation.

### Kaempferol hampers the metastasis of gastrointestinal cancer

**Figure 8:**
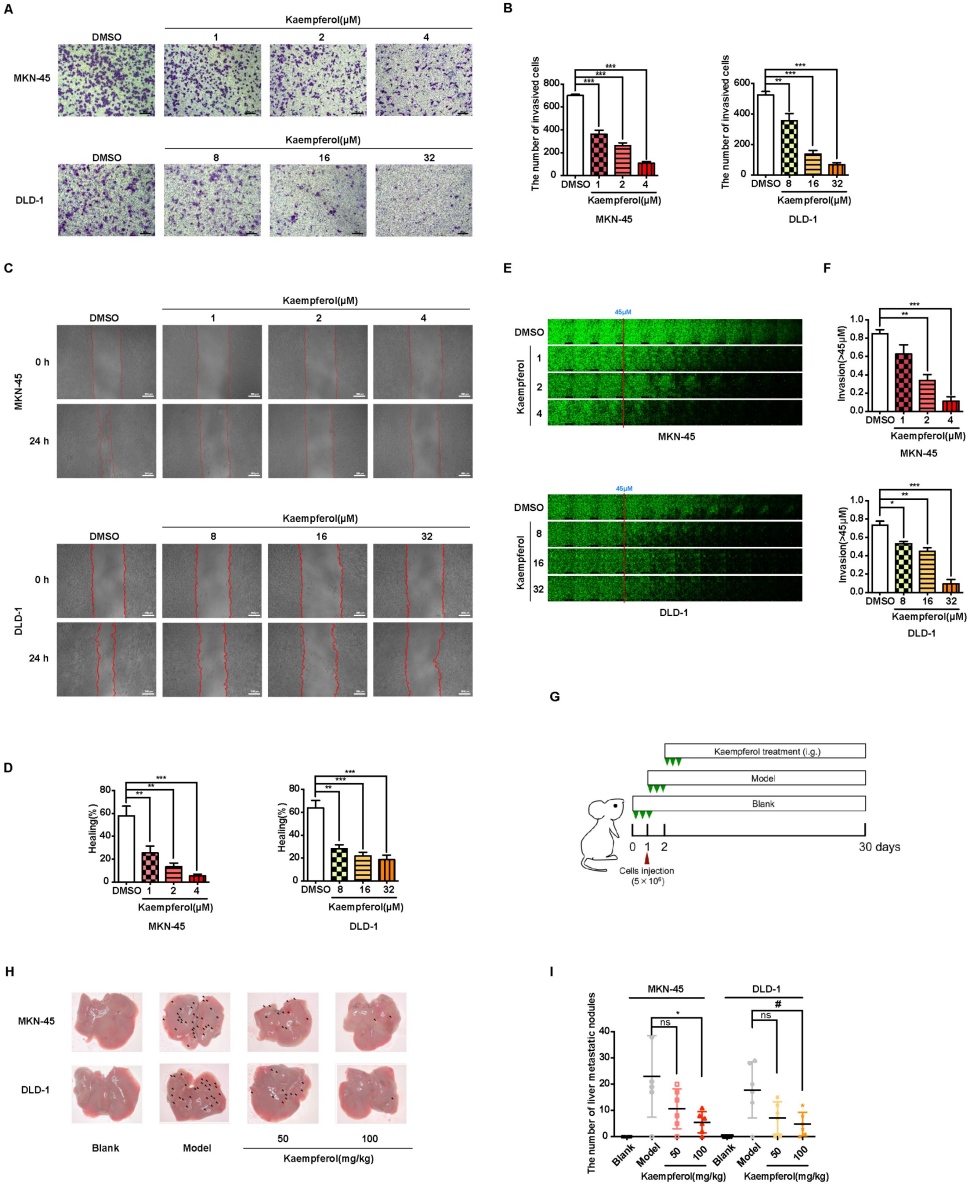
Kaempferol inhibits gastrointestinal cancer metastasis both *in vitro* and *in vivo*. (A) Representative transwell assay images showing the migration of MKN-45 and DLD-1 cells treated with various concentrations of kaempferol. Random visual fields were selected for analysis. Scale bar: 500 μm. (B) Bar graphs showing the number of migrated MKN-45 and DLD-1 cells treated with different concentrations of kaempferol. Data are expressed as means ± SD, **P < 0.01, ***P < 0.001 (vs. DMSO group). (C) Representative wound healing assay images of MKN-45 and DLD-1 cells treated with different concentrations of kaempferol. (D) Healing rates of MKN-45 and DLD-1 cells treated with kaempferol at different concentrations. **P < 0.01, ***P < 0.001 (vs. DMSO group). (E) Inverted invasion assay evaluating the invasion capabilities of MKN-45 and DLD-1 cells in the presence of different concentrations of kaempferol. Scale bar: 250 μm. (F) Quantification of relative invasion of MKN-45 and DLD-1 cells over 45 μm (n = 3). Data are expressed as means ± SD, *P < 0.05, **P < 0.01, ***P < 0.001 (vs. DMSO group). (G) Schematic of the animal study: 5 × 10^6^ MKN-45 or DLD-1 cells were injected into BALB/c nude mice, and 50 mg/kg and 100 mg/kg of kaempferol were administered intraperitoneally starting on day 2 post-injection (n = 8 per group). (H, I) Liver metastasis analysis in mice engrafted with MKN-45 or DLD-1 cells. Data are expressed as means ± SD, *P < 0.05 (vs. MKN-45 model group), ^#^P < 0.05 (vs. DLD-1 model group).

Kaempferol, a flavonoid abundantly found in *Rhizoma Kaempferiae* and nearly 400 varieties of fruits and vegetables, holds significant potential in the development of anti-cancer therapies 30. To investigate its effects on gastrointestinal cancer, a series of *in vitro* experiments were conducted. The results demonstrate that kaempferol notably suppresses the migration, invasion, and metastasis of gastrointestinal cancer cells in a dose-dependent manner (Figures 8A-8F). Furthermore, after injecting MKN-45 and DLD-1 cells into BALB/c nude mice, data revealed a substantial reduction in the number of metastatic nodules on liver surfaces in tumor xenografts treated with kaempferol (Figures 8G-8H). Notably, no significant changes were observed in the number of liver metastases at a dosage of 50 mg/kg compared to the control, but a marked reduction was seen with 100 mg/kg (Figure 8I). To verify whether this effect extends to other gastrointestinal cancer cell lines, *in vivo* studies using the BGC-823 gastric cancer cell line were performed. BGC-823-RFP cells were intravenously injected into mice, and consistent with previous findings, kaempferol significantly reduced lung metastases (Supplementary Figure 6A). Both 50 mg/kg and 100 mg/kg doses significantly decreased the fluorescence intensity of metastatic cells in mice compared to the control (Supplementary Figure 6B). Additionally, while kaempferol did not induce apoptosis in MKN-45 and DLD-1 cells, it did reduce ROS levels to a certain degree (Supplementary Figures 6C-6D). Moreover, kaempferol impaired the formation of filopodia and lowered the expression of related proteins (Supplementary Figures 6E-6G). Collectively, these results highlight kaempferol's critical role in inhibiting the metastasis of gastrointestinal cancers both *in vitro* and *in vivo*.

## Discussion

**Figure 9:**
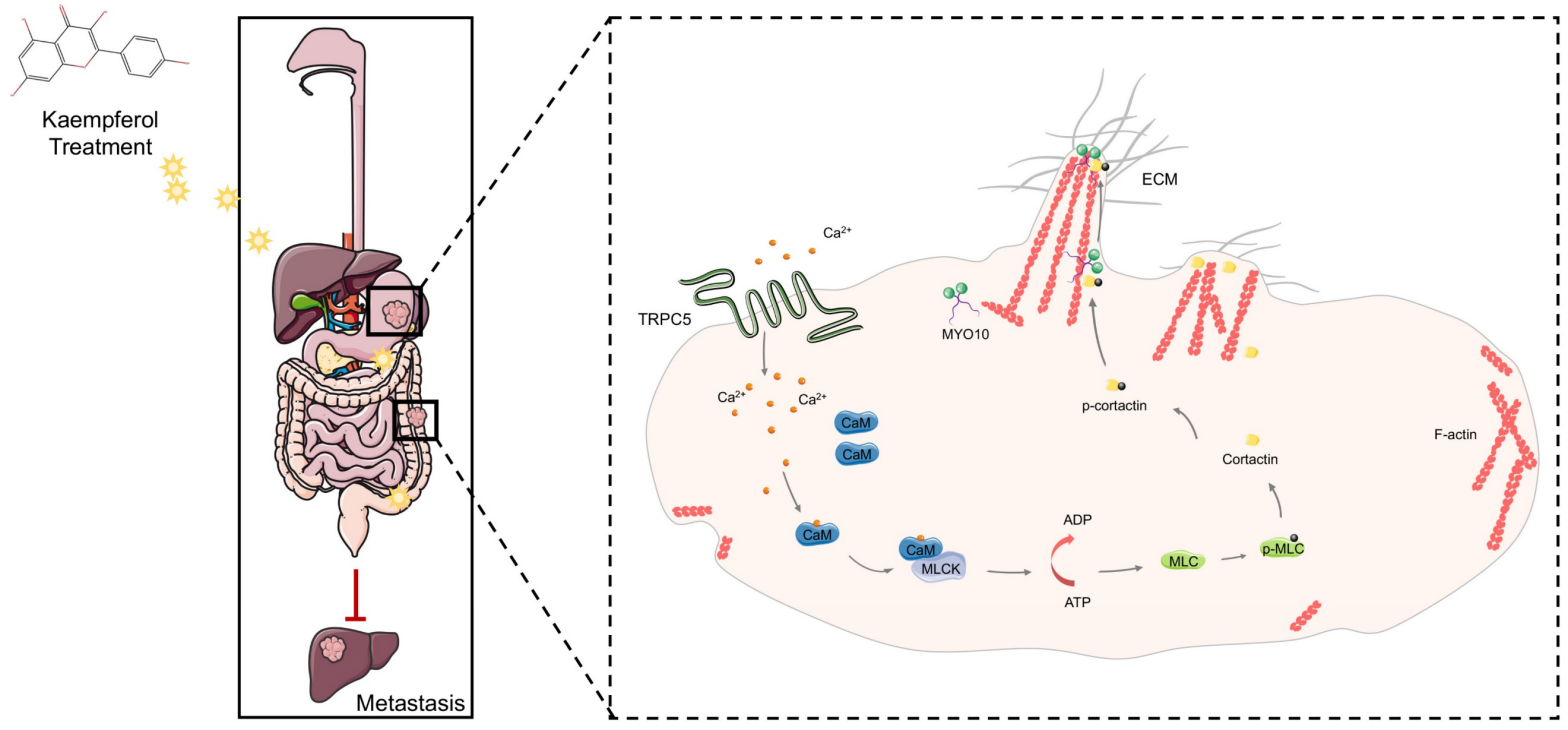
**TRPC5 drives filopodia formation by activating the ATP/p-MLC/p-Cortactin signaling axis.** Activation of TRPC5 channels in gastrointestinal cancer cells triggers calcium ion influx, leading to the formation of the Ca^2+^-CaM complex, which promotes ATP-dependent MLC phosphorylation. This phosphorylation enhances the activation and localization of cortactin at the filopodia tips, regulating their formation and stabilization. Kaempferol, a promising natural small-molecule TRPC5 inhibitor, disrupts this process by targeting TRPC5, thereby inhibiting filopodia formation and stabilization, ultimately suppressing the metastasis of gastrointestinal cancer.

This study reveals that TRPC5 is functionally expressed in gastrointestinal cancer and is negatively associated with patient prognosis. Our findings highlight that TRPC5 activation in gastrointestinal cancer cells promotes calcium ion influx, leading to the formation of the Ca^2+^-CaM complex, both of which contribute to filopodia formation. The enhancement of filopodia by TRPC5 is mediated through the ATP/p-MLC/p-cortactin signaling pathway. Notably, pharmacological inhibition of TRPC5 by kaempferol or ML-204 reduces intracellular Ca^2+^ influx, thereby impairing MYO10-mediated transport of p-cortactin to the filopodia tips, ultimately suppressing metastasis in gastrointestinal cancer cells (Figure 9).

Malignant tumors are highly invasive, and metastasis is a leading cause of death in patients with gastrointestinal cancer. Understanding the key drivers of metastasis, particularly those linked to environmental factors, could uncover potential therapeutic targets. TRP channels, including TRPC5, are non-selective cation channels that respond to transient changes in temperature, mechanical stress, and chemical stimuli, influencing cellular behavior 51. Abnormal TRP expression has been widely implicated in the promotion of metastasis 52. Our research demonstrates that TRPC5 is overexpressed in clinical samples of STAD, COAD, and various gastrointestinal cancer cell lines, with its expression negatively correlated with overall patient survival. The tendency of MKN-45 and BGC-823 cell lines to metastasize to various organ sites is a notable phenomenon that warrants further investigation. Additionally, the expression of TRPC5, as indicated by the increased intracellular Ca^2+^ concentration, provides insight into its functional role. Given the lack of specific TRPC5 agonists, Gd^3+^ was employed in this study to activate TRPC5 channels. It is well-established that Gd^3+^ effectively activates TRPC5 channels, and its stimulation does not alter cell current or calcium ion levels in cells that do not express TRPC5. This confirms the feasibility of using Gd^3+^ as a TRPC5 activator. Both *in vitro* and *in vivo* evidence suggests that the deletion and functional inhibition of TRPC5 can impede gastrointestinal cancer metastasis, highlighting TRPC5 as a potential therapeutic target for managing cancer metastasis.

While directed motility is a characteristic of all cells during development, tissue repair, and regeneration, the shift from a benign to malignant phenotype is marked by the cancer cells' acquired invasive capacity—a hallmark of aggressive cancers. Substantial evidence shows that cancer cells undergo local invasion, distant metastasis, and colonization by generating invasive structures such as filopodia, lamellipodia, and other pseudopodia during metastasis 38. Notably, L-type calcium channels have been implicated in promoting metastasis by stabilizing filopodia 9. Given this, we hypothesized that TRPC5, as a non-selective Ca^2+^ channel, may be relevant to cancer cell motility and filopodia formation. Analysis using multiple databases revealed that TRPC5 plays a critical role in regulating cancer cell motility Subsequent experiments confirmed that TRPC5 enhances filopodia formation by activating the ATP/p-MLC/p-cortactin signaling axis. Additionally, MLCK has been reported to maintain TRPC5 channel activity, suggesting a potential positive feedback loop between MLCK and TRPC5. These results provide insight into why TRPC5 overexpression is negatively correlated with patient prognosis.

According to species evolution theory, plant-derived TCM components are produced in response to environmental stress and can interact with biological targets to exert pharmacological effects 53. This led us to explore active TCM components that may inhibit TRPC5 function, with the goal of developing anti-cancer drugs targeting gastrointestinal cancer. Fortunately, kaempferol, derived from Kaempferiae Rhizoma, was identified, which significantly inhibits TRPC5-mediated calcium influx and blocks the downstream effects of TRPC5 activation. The DARTS and CETSA assays further suggest that kaempferol's inhibitory effect on TRPC5 may result from its direct binding to the channel. Multiple studies have already highlighted kaempferol's broad potential in preventing and treating various cancers 54, 55. Our findings demonstrate that kaempferol exerts significant anti-metastatic effects on gastrointestinal cancer both *in vitro* and *in vivo*, likely through TRPC5 inhibition. However, the current evidence for kaempferol's binding to TRPC5 remains indirect, warranting further studies using more direct approaches, such as biotin-labeled drug probes. Although our results emphasize kaempferol's key role in TRPC5 inhibition, the possibility of compensatory mechanisms involving other TRP channels cannot be ruled out. Additionally, cancer metastasis is influenced by numerous factors, including reactive oxygen species production, angiogenesis, metabolic reprogramming, and immune evasion. In this study, kaempferol was identified through its targeting of TRPC5, shedding light on its potential mechanism against cancer metastasis. That said, we cannot exclude the possibility that kaempferol may inhibit cancer metastasis through other pathways, and further investigations will be required to explore this in depth. In conclusion, this study highlights TRPC5 as a promising target for gastrointestinal cancer treatment, shedding light on the mechanisms by which filopodia formation facilitates metastasis. These findings offer a novel and reliable therapeutic approach for inhibiting cancer spread. Moreover, kaempferol, a TCM-derived compound, is identified as a natural small-molecule inhibitor of TRPC5, effectively reducing metastasis. Given kaempferol's widespread availability in both TCM and various fruits and vegetables, it holds significant potential as a readily accessible anti-metastatic agent for gastrointestinal cancer. Additionally, the aberrant expression of various thermo-TRPs in multiple tumor types suggests that screening TCM compounds based on their “cold/hot” properties may offer further opportunities to target alternative thermo-TRPs for cancer therapy.

## Supplementary Material

Supplementary figures and tables.
