# Supplementary material for: Pharmacological manipulation of TRPC5 by kaempferol attenuates metastasis of gastrointestinal cancer via inhibiting calcium involved in the formation of filopodia
Source: Int J Biol Sci. 2024 Sep 16;20(12):4922–40. doi: 10.7150/ijbs.87829 (PMC11414399; doi:10.7150/ijbs.87829)
Supplement: Supplementary file 1 — Supplementary figures and tables. [file ijbsv20p4922s1.pdf]

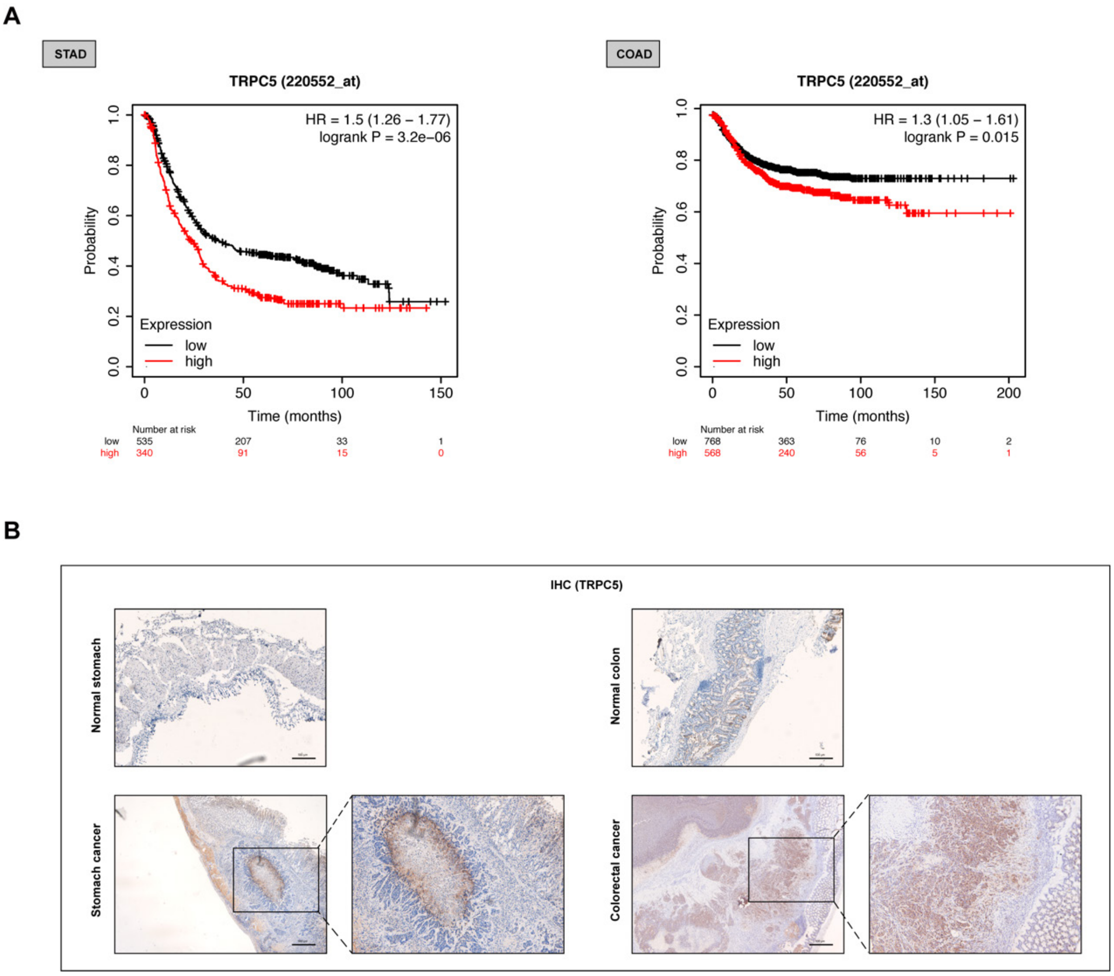

Supplementary Fig. 1

TRPC5 is negatively correlated with prognosis of patients with gastrointestinal cancer. (A) Kaplan–Meier survival curves for the gastric and colorectal patients with high- (red) and low- (black) median TRPC5 copy number estimates (n = 875 for STAD, n = 1336 for COAD). (B) Representative TRPC5 IHC staining of normal tissues and primary cancers (gastric and colon) (n = 5).

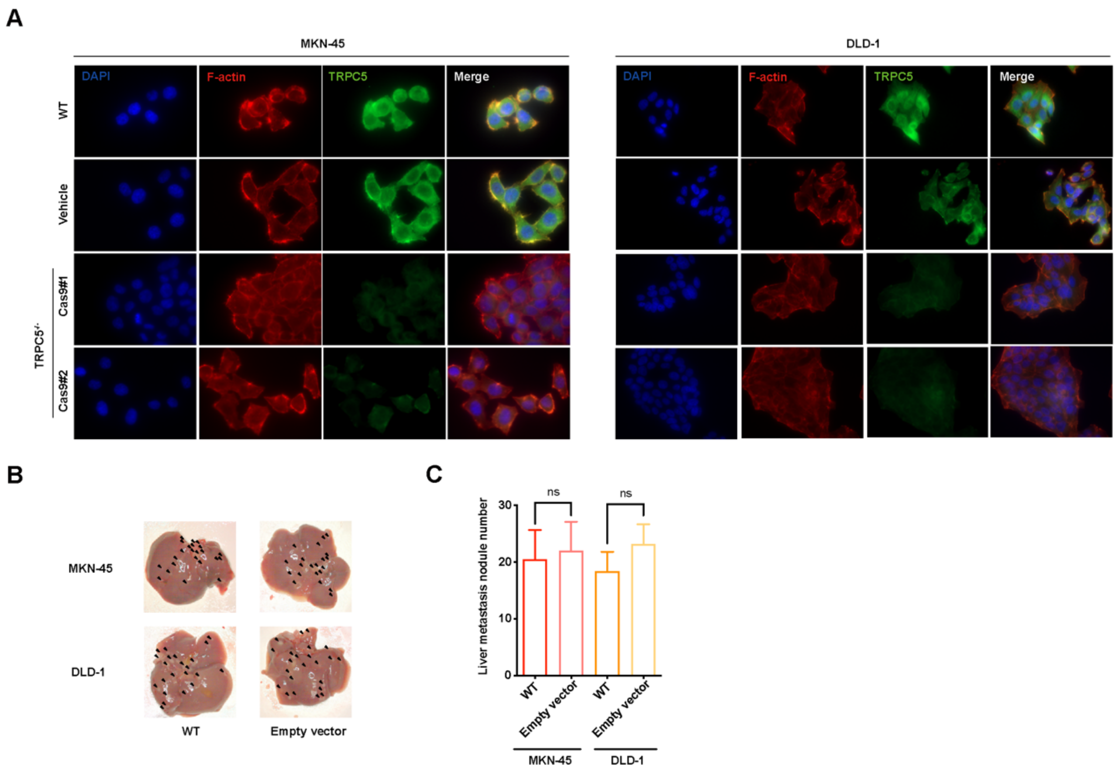

Supplementary Fig. 2

Effects of TRPC5 expression on gastrointestinal cancer. (A) Representative IF images of MKN-45 and DLD-1 stained with TRPC5 (blue, nuclei; red, F-actin; green, TRPC5). Scale bar, 50 μm. (B and C) Liver metastasis analysis after mice engrafted with MKN-45 and DLD-1 cells (WT or Empty vector) at day 30. The data are expressed as means ± SD.

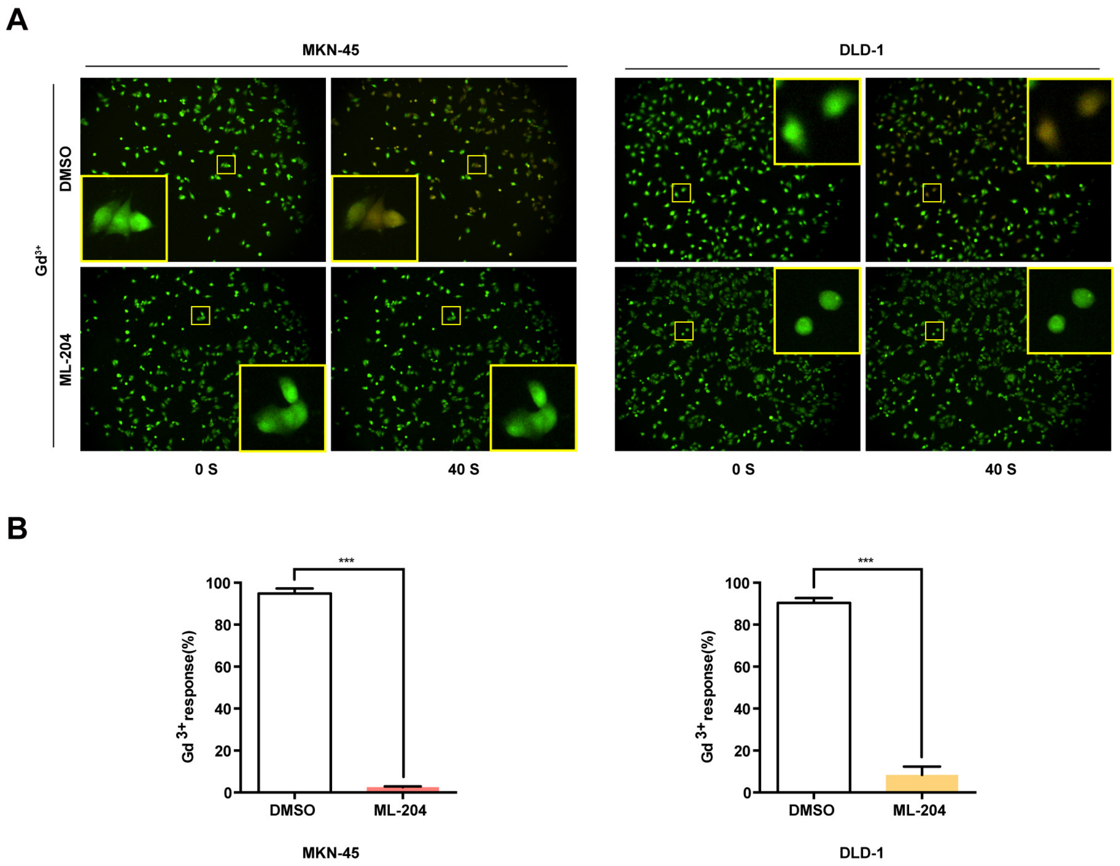

Supplementary Fig. 3

ML-204 inhibits TRPC5-mediated calcium influx. (A) The Ca^{2+} imaging results with representative images of calcium responses in MKN-45 and DLD-1 cells evoked by 50 μM Gd^{3+} at 0 s and 40 s pretreated with DMSO or ML-204 (8 μM). (B) Ratios of Gd^{3+} response in MKN-45 and DLD-1 cells evoked by 50 μM Gd^{3+} pretreated with DMSO or ML-204 (8 μM). The data are expressed as means ± SD, ***$P < 0.001$ (versus DMSO group).

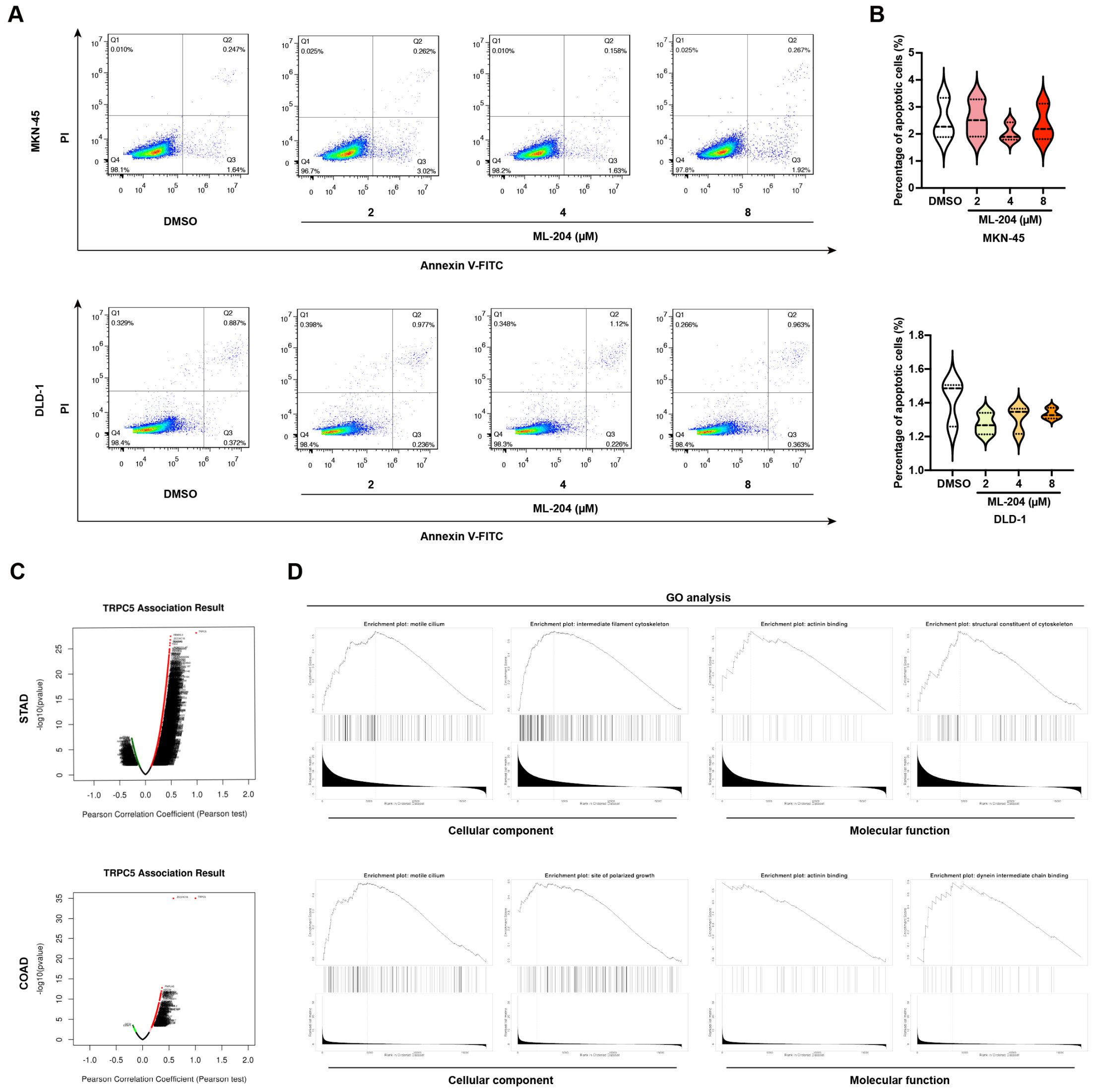

Supplementary Fig. 4

Biological processes associated with TRPC5 in gastric cancer and colorectal cancer. (A) Cancer cell apoptosis in each group was determined by flow cytometry analysis. (B) The quantified data of cancer cell apoptosis (versus DMSO group). The data are expressed as means ± SD. (C) Volcanic map of genes closely related to TRPC5 function in gastric and colorectal cancer. (D) GO analysis of TRPC5-associated genes on cellular component and molecular function.

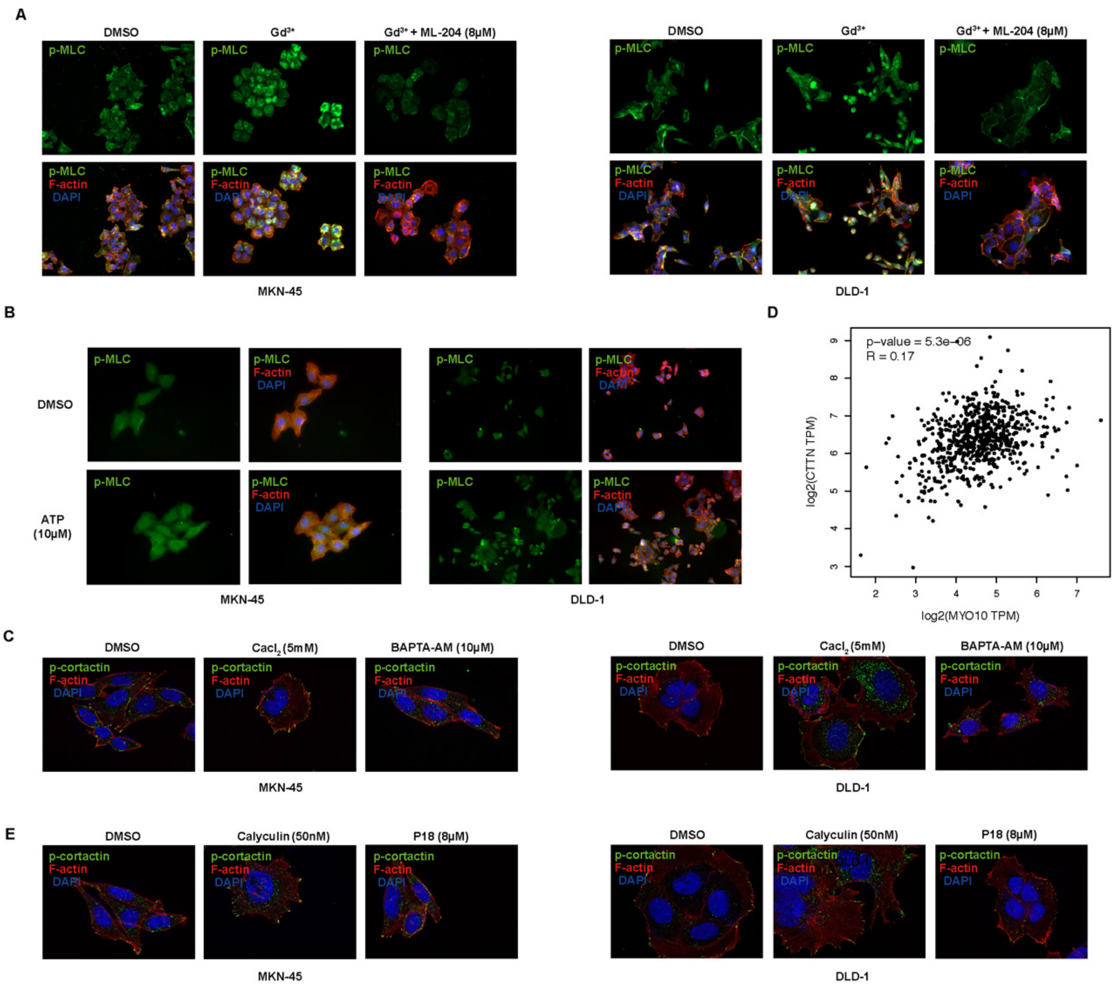

Supplementary Fig. 5

Mechanisms of TRPC5 regulating filopodia formation. (A) Representative p-MLC IF staining images of MKN-45 and DLD-1 cells treated with Gd^{3+} and ML-204 (8 μM) (blue, nuclei; red, F-actin; green, p-MLC). (B) Representative p-MLC IF staining images of MKN-45 and DLD-1 cells treated with ATP (10 μM) (blue, nuclei; red, F-actin; green, p-MLC). (C) Representative p-cortactin IF staining images of MKN-45 and DLD-1 cells treated with $Cacl_2$ (5 mM) and BAPTA-AM (10 μM) (blue, nuclei; red, F-actin; green, p-cortactin). (D) Correlation analysis between MYO10 and CTTN genes. (E) Representative p-cortactin IF staining images of MKN-45 and DLD-1 cells treated with Calyculin (50 nM) and P18 (8 μM) (blue, nuclei; red, F-actin; green, p-cortactin).

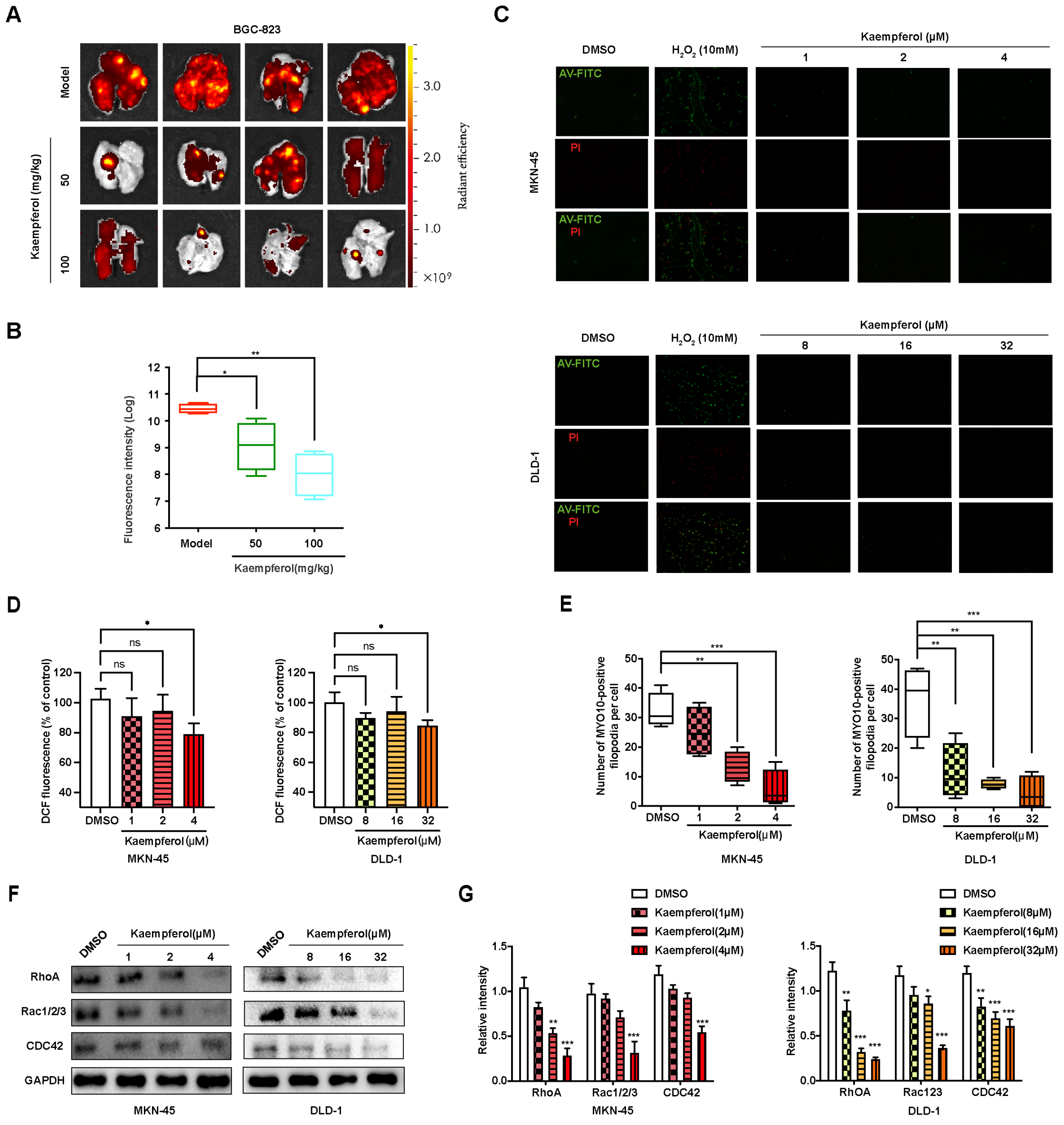

Supplementary Fig. 6

Effects and mechanisms of kaempferol on gastrointestinal cancer. (A) Lung metastasis of BGC-823-RFP cells treated with kaempferol (50mg/kg and 100mg/kg) in the experimental metastasis model (n=6). (B) Quantification of fluorescence intensity of BGC-823-RFP cells treated with kaempferol (50mg/kg and 100mg/kg) (versus model group) in the experimental metastasis model. (C) Representative apoptosis fluorescence images of MKN-45 and DLD-1 cells treated with kaempferol (red, PI; green, Annexin V-FITC). (D) Quantification of fluorescence intensity of ROS in MKN-45 and DLD-1 cells treated with kaempferol (versus DMSO group). (E) The number of MYO10-positive filopodia in the MKN-45 and DLD-1 cells treated with kaempferol

(versus DMSO group). (F-G) Immunoblot analysis of RhoA, Rac1/2/3 and CDC42 protein expression in the MKN-45 and DLD-1 cells treated with kaempferol (versus DMSO group). The data are expressed as means ± SD, *P < 0.05, **P < 0.01, ***P < 0.001.

Table S1. Primer of Genes

Genes	Forward	Reverse
TRPC5	5'-ACTCTGATTGCGGAAGCACT-3'	5'-GGATCCCCTTGCAGTTGTTA-3'
GAPDH	5'-GGTTGTCTCCTGCGACTTCA-3'	5'-TGGTCCAGGGTTTCTTACTCC-3'

Table S2. Summary table of clinic samples

Sample ID	Cancer type	Sample information
S1415513	STAD	Moderately differentiated adenocarcinoma
S1505116	STAD	Infiltrated poorly differentiated adenocarcinoma
S1517162	STAD	Poorly differentiated adenocarcinoma
S1507716	STAD	Moderately and poorly differentiated adenocarcinoma
S1517162	STAD	Poorly differentiated adenocarcinoma
20120565	COAD	Accompanied metastasis
20120637	COAD	Accompanied metastasis
20120648	COAD	Accompanied metastasis
20120676	COAD	Accompanied metastasis
20120691	COAD	Accompanied metastasis

20120692	COAD	Accompanied metastasis
20120693	COAD	Accompanied metastasis
20120694	COAD	Accompanied metastasis
20121013	COAD	Accompanied metastasis
20121043	COAD	Accompanied metastasis
20121053	COAD	Accompanied metastasis
20121071	COAD	Not accompanied metastasis
20121081	COAD	Accompanied metastasis
20121147	COAD	Accompanied metastasis
20121201	COAD	Accompanied metastasis
20121216	COAD	Accompanied metastasis
20121290	COAD	Accompanied metastasis
20120193	COAD	Accompanied metastasis
20181683	COAD	Accompanied metastasis
20181793	COAD	Accompanied metastasis
20181889	COAD	Accompanied metastasis
